# The metabolic hormone leptin promotes the function of T_FH_ cells and supports vaccine responses

**DOI:** 10.1038/s41467-021-23220-x

**Published:** 2021-05-24

**Authors:** Jun Deng, Qian Chen, Zhian Chen, Kaili Liang, Xin Gao, Xiaohui Wang, Fadzai V. Makota, Hong Sheng Ong, Yanmin Wan, Kaiming Luo, Dongcheng Gong, Xiang Yu, Sarina Camuglia, Qunxiong Zeng, Tao Zhou, Feng Xue, Jing He, Yunbo Wei, Fan Xiao, Jianyang Ma, Danika L. Hill, Wim Pierson, Thi H. O. Nguyen, Haibo Zhou, Yan Wang, Wei Shen, Lingyun Sun, Zhanguo Li, Qiang Xia, Kun Qian, Lilin Ye, Steven Rockman, Michelle A. Linterman, Katherine Kedzierska, Nan Shen, Liwei Lu, Di Yu

**Affiliations:** 1 China-Australia Centre for Personalised Immunology, Renji Hospital, Shanghai Jiao Tong University School of Medicine, Shanghai, China; 2 Department of Pathology and Shenzhen Institute of Research and Innovation, The University of Hong Kong, Hong Kong, China; 3 State Key Laboratory of Oncogenes and Related Genes, Shanghai Cancer Institute, Renji Hospital, Shanghai Jiao Tong University School of Medicine, Shanghai, China; 4 Department of Ophthalmology, Shanghai General Hospital, Shanghai Jiao Tong University School of Medicine, Shanghai, China; 5 Department of Immunology and Infectious Disease, John Curtin School of Medical Research, Australian National University, Canberra, ACT Australia; 6 The University of Queensland Diamantina Institute, Translational Research Institute, Faculty of Medicine, The University of Queensland, Brisbane, QLD Australia; 7 Department of Infectious Diseases, Huashan Hospital, Fudan University, Shanghai, China; 8 Department of Radiology, Shanghai Public Health Clinical Center, Fudan University, Shanghai, China; 9 Shanghai Institute of Rheumatology, Renji Hospital, Shanghai Jiao Tong University School of Medicine, Shanghai, China; 10 Seqirus, Parkville, VIC Australia; 11 Department of Liver Surgery, Renji Hospital, Shanghai Jiao Tong University School of Medicine, Shanghai, China; 12 Department of Rheumatology and Immunology, Peking University People’s Hospital, Beijing, China; 13 Laboratory of Immunology for Environment and Health, Shandong Analysis and Test Center, Qilu University of Technology (Shandong Academy of Sciences), Jinan, China; 14 Lymphocyte Signaling and Development, Babraham Institute, Cambridge, UK; 15 Department of Microbiology and Immunology, University of Melbourne, Peter Doherty Institute for Infection and Immunity, Parkville, VIC Australia; 16 Department of Laboratory Medicine, Renji Hospital, Shanghai Jiao Tong University School of Medicine, Shanghai, China; 17 Department of Rheumatology and Immunology, The Affiliated Drum Tower Hospital of Nanjing University Medical School, Nanjing, China; 18 School of Biomedical Engineering, Shanghai Jiao Tong University, Shanghai, China; 19 Institute of Immunology, Third Military Medical University, Chongqing, China; 20 Chongqing International Institute for Immunology, Chongqing, China

**Keywords:** Lymphocyte differentiation, Antibodies, Vaccines

## Abstract

Follicular helper T (T_FH_) cells control antibody responses by supporting antibody affinity maturation and memory formation. Inadequate T_FH_ function has been found in individuals with ineffective responses to vaccines, but the mechanism underlying T_FH_ regulation in vaccination is not understood. Here, we report that lower serum levels of the metabolic hormone leptin associate with reduced vaccine responses to influenza or hepatitis B virus vaccines in healthy populations. Leptin promotes mouse and human T_FH_ differentiation and IL-21 production via STAT3 and mTOR pathways. Leptin receptor deficiency impairs T_FH_ generation and antibody responses in immunisation and infection. Similarly, leptin deficiency induced by fasting reduces influenza vaccination-mediated protection for the subsequent infection challenge, which is mostly rescued by leptin replacement. Our results identify leptin as a regulator of T_FH_ cell differentiation and function and indicate low levels of leptin as a risk factor for vaccine failure.

## Abstract (web summary)

T follicular helper (TFH) cell numbers are increased after vaccination and fewer of these cells might result in reduced vaccine responses. Here the authors show in mice and humans that leptin promotes T_FH_ differentiation and that low leptin levels can impair T_FH_ response to vaccines and virus protection in mice.

## Introduction

Follicular helper T (T_FH_) cells constitute a specialised CD4^+^ T-cell subset that is essential for supporting humoral immunity^1,2^. The importance of T_FH_ cells in health is evidenced by the identification of genetic mutations in genes encoding proteins critical to mounting T_FH_-cell-dependent humoral responses: inducible co-stimulatory receptor ICOS, SLAM-associated signal adaptor protein (SAP), cytokine receptors for IL-12 and IL-21, and signal transducer and activator of transcription 3 (STAT3). Loss-of-function mutations in these genes impair the generation of functional T_FH_ cells and lead to defective humoral immunity^1,3,4^. On the other hand, excessive T_FH_ generation and function promote the production of autoantibodies and the development of autoimmune diseases^1,4,5^. Studies suggest that aberrant T_FH_ generation in autoimmune disease is likely driven by continuous (auto)antigen stimulation and the upregulation of T_FH_-inducing cytokines, such as IL-6^5–8^. Between these two extreme ends of the spectrum of T_FH_ cell function, we know very little about how the function of T_FH_ cells is regulated in the general population.

As one of modern medicine’s greatest successes, vaccinations against childhood and adult infections have saved millions of lives. However, it is not always effective in all individuals. Even in a healthy general population, vaccination is often limited by poor responses in a significant portion of people^9,10^. For example, the clinical efficacy of a standard dose of non-adjuvanted influenza vaccine was 70–90% in young adults but dropped sharply to only 17–53% in the elderly (aged ≥ 65 years)^11^. One key factor limiting vaccine responses is the activity of T_FH_ cells. We and others have reported that T_FH_ cells are induced early following vaccination^7,12–14^, and the frequency of functional T_FH_ cells correlates with the strength of B-cell responses and protective antibody production^12,13,15–17^. Therefore, it is conceivable that variable vaccine responses among the healthy population might be regulated by individual activities of T_FH_ cells. What determines the T_FH_ activity among the general population?

Studies have highlighted the major influence that the metabolic state can exert on the immune system^18–20^. Employing a systemic approach, the multiscale, multifactorial response network, Li et al. revealed that metabolic phenotypes are tightly coupled with vaccine immunity in healthy individuals^21^. As an adipokine that is abundantly secreted by adipose tissue, the metabolic hormone leptin broadly regulates many immune cell types of both innate and adaptive immune systems^19,22,23^. Notably, leptin levels vary drastically (range over 10-fold) in healthy individuals^15^, but the function of leptin in regulating T_FH_ cells and vaccine responses remains unclear. We, therefore, investigated whether leptin is a natural regulator of T_FH_ function in healthy individuals and whether it plays a role in determining vaccine response.

In this study, we show that low levels of serum leptin in both young and elderly healthy groups are associated with reduced antibody responses to influenza and hepatitis B virus (HBV) vaccines. Leptin promotes the differentiation and function of both human and mouse T_FH_ cells in culture and is required to support T_FH_ function and effective humoral immunity to infection, immunisation and vaccination in mice. The mechanism of action of leptin is mediated partially through the activation of Stat3 and the mechanistic target of rapamycin (mTOR) pathways. Our results suggest leptin is a physiological regulator of T_FH_ function and insufficient leptin might serve as a biomarker to identify the risk for low vaccine efficacy.

## Results

### Low leptin levels are associated with reduced vaccine responses

**Fig. 1:**
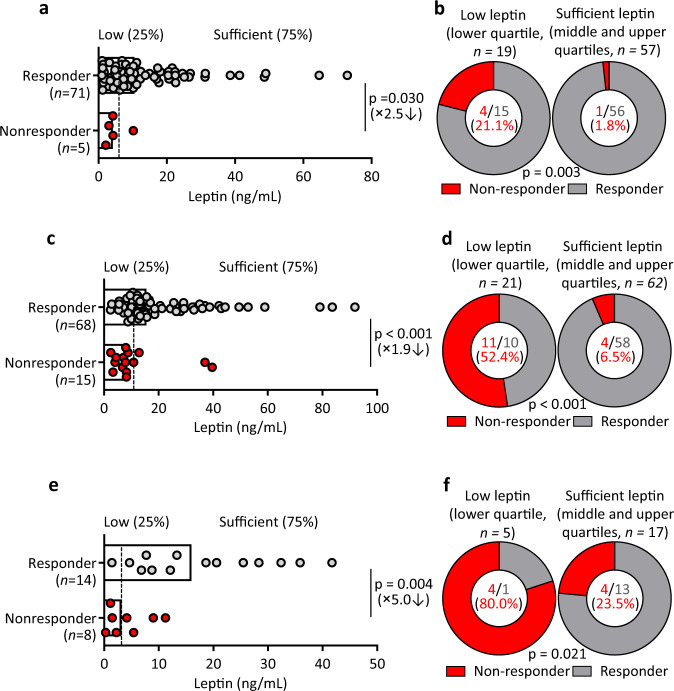
Low leptin levels are associated with lower antibody responses after vaccination in the general population. **a** Comparison of serum leptin levels between responders (*n* = 71, grey) and non-responders (*n* = 5, red) in healthy adults (18–60 years, *n* = 76) immunized with the influenza vaccine. Dotted line indicates healthy individuals with low leptin levels (lower 25%, <4.8 ng/mL) and sufficient leptin levels (higher 75%, ≥4.8 ng/mL). **b** Comparison of response rates between low leptin (*n* = 19) and sufficient leptin (*n* = 57) groups. **c** Comparison of serum leptin levels between responders (*n* = 68, grey) and non-responders (*n* = 15, red) in healthy elderly adults (>65 years, *n* = 83) immunized with the influenza vaccine. Dotted line indicates healthy individuals with low leptin levels (lower 25%, <8.8 ng/mL) and sufficient leptin levels (higher 75%, ≥8.8 ng/mL). **d** Comparison of response rates between low leptin (*n* = 21) and sufficient leptin (*n* = 62) groups. **e** Comparison of serum leptin levels between responders (*n* = 14, grey) and non-responders (*n* = 8, red) in healthy young adults (18–25 years, *n* = 22) immunized with the HBV vaccine. Dotted line indicates healthy individuals with low leptin levels (lower 25%, <3.2 ng/mL) and sufficient leptin levels (higher 75%, ≥3.2 ng/mL). **f**, Comparison of response rates between low leptin (*n* = 8) and sufficient leptin (*n* = 14) groups. Data from individuals (dots) and the median values (bars) are shown for **a**, **c** and **e**; Mann–Whitney test was used for analysis (**a**, **c**, **e**). Percentages and numbers are shown for **b**, **d** and **f**; Chi-Square tests were used for analysis (**b**, **d**, **f**). Detailed demographics of each cohort are shown in Supplementary Tables 1-3. For correlation of leptin levels and antibody responses see Supplementary Fig. 1.

We first examined the relationship between serum leptin levels and the immune response in healthy adults (aged 18–60 years, *n* = 76) immunised with a standard dose of non-adjuvanted trivalent influenza vaccine^14,24^. We classified individuals into responder group (HI titer fold-change to any one of the three strains at baseline and after vaccination ≥2, *n* = 71) and non-responder group (HI titer to all three strains <20 and HI titer fold-change to all three strains <2, *n* = 5) (Supplementary table 1). We found the serum leptin levels of the non-responder group showed about 2.5-fold reduction compared to those of the responder group: median, 4.2 vs 10.5 ng/mL (*P* = 0.03, Student’s *t*-test; Fig. 1a). The lower leptin levels in non-responders than responders suggested leptin insufficiency as a risk factor for vaccine response. Therefore, individuals were divided into the group with relatively low levels of leptin (lower quartile, *n* = 19) and sufficient levels of leptin (middle and upper quartiles, *n* = 57). More strikingly, we observed that non-responders were highly enriched in the low leptin group: the non-response rate of 21.1% in the low leptin group vs 1.8% in the sufficient leptin group (*P* = 0.003, Chi-Square test; Fig. 1b).

The vaccine efficacy significantly drops in the elderly group^11^. This promoted us to conduct a similar analysis in a group of healthy elderly adults (aged ≥ 65 years, *n* = 83) immunised with a standard dose of non-adjuvanted trivalent influenza vaccine. We observed that low leptin levels were again associated with poor antibody responses after vaccination in healthy elderly adults. The serum leptin levels of the non-responder group were significantly lower than those of the responder group: median, 8.0 vs 15.5 ng/mL, *P* < 0.001, Student’s *t*-test; Supplementary table 2, Fig. 1c). The individuals with relatively low levels of leptin (the lower quartile, *n* = 21) were highly enriched with non-responders: the non-response rate was 52.4% in the low leptin group vs 6.5% in the sufficient leptin group (*P* < 0.001, Chi-Square test; Fig. 1d).

To test whether the association between leptin levels and vaccination responses was a common phenomenon for a different vaccine, we next examined a group of young adults (aged 18–25 years, *n* = 22) with low serological anti-HBs (<100 mIU/mL) who were re-immunised with the vaccine for HBV (Supplementary table 3). The non-responder group (serum antibody titre <200 mIU/mL after primary immunisation, *n* = 8) showed about 5.0-fold lower leptin levels than the responder group (serum antibody titter fold changes ≥2, *n* = 14): median 3.2 vs 16.0 ng/mL (*P* = 0.004, Student’s *t*-test; Fig. 1e). The non-response rate was 80.0% in the low leptin group (the lower quartile, *n* = 5) and 23.5% in the sufficient leptin group (*n* = 17) (*P* = 0.021, Chi-Square test; Fig. 1f). These results demonstrated that in different vaccination and different age groups, non-responders showed lower leptin levels than responders, suggesting that low levels of leptin could be a limiting factor for effective vaccine responses or indicate a relationship between vaccine responses and systemic metabolic state, since serum leptin concentrations correlate with the percentage of body fat^15,25^.

### Leptin potentiates the differentiation and function of human T_FH_ cells

**Fig. 2:**
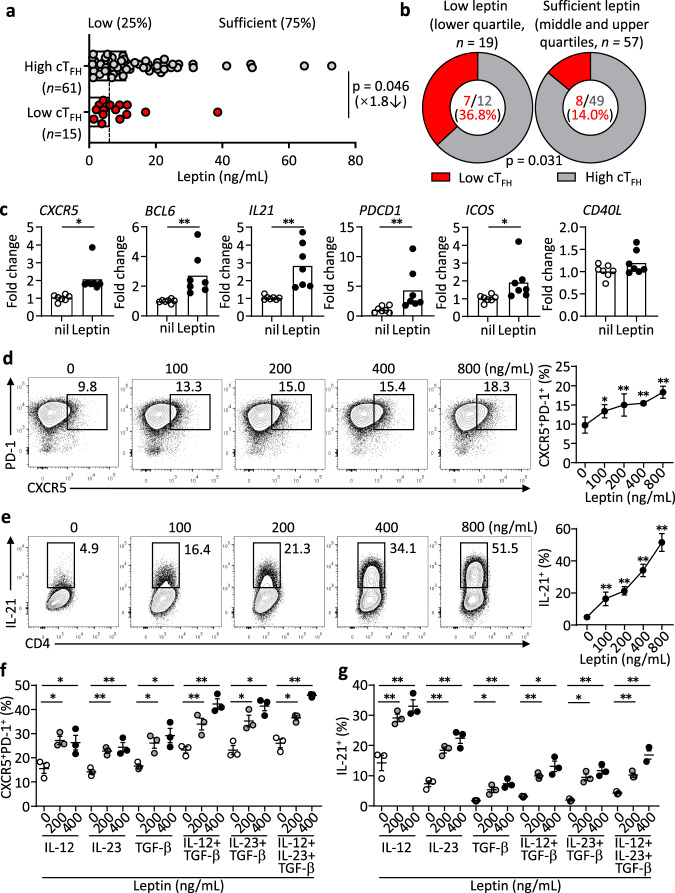
Leptin potentiates the differentiation and function of human T_FH_ cells. **a** Comparison of serum leptin levels between healthy individuals with T_FH_ increase (fold changes of T_FH_ cells, the ratio of day 7/day 0 > 1) (*n* = 61, grey) and healthy individuals without T_FH_ increase (fold changes of T_FH_ cells, the ratio of day 7/day 0 ≤ 1) (*n* = 15, red) in healthy adults (18–60 years, *n* = 76) immunized with the influenza vaccine (Supplementary Table 4). Dotted line indicates healthy individuals with low leptin levels (lower 25%) and sufficient leptin levels (higher 75%). **b** Comparison of T_FH_ changes and vaccine response in low leptin group (*n* = 19) and sufficient leptin group (*n* = 57). **c** Real-time PCR analysis of T_FH_-related genes in naïve CD4^+^ T cells from PBMC of healthy controls with anti-CD3/CD28 activation for 3 days, and further treated with leptin for 12 h (*CXCR5*: *P = 0.0176, *Bcl6*: **P = 0.0046, *IL21*: **P = 0.0085, *PDCD1*: **P = 0.0050, *ICOS*: **P = 0.0344) (*n* = 7). **d**, **e** Representative FACS plots and statistics showing the differentiation of CXCR5^+^PD-1^+^ T_FH_ cells (**d**) (100 ng/mL: *P = 0.0257, 200 ng/mL: **P = 0.0012, 400 ng/mL: **P = 0.0005, 800 ng/mL: **P < 0.0001), and the secretion of IL-21 (**e**) (100 ng/mL: **P = 0.0004, 200 ng/mL: **P < 0.0001, 400 ng/mL: **P < 0.0001, 800 ng/mL: **P < 0.0001) from purified human naïve CD4^+^ T cells stimulated with anti-CD3/CD28 and indicated concentrations of leptin for 4 days (dots, *n* = 5; bars, mean ± SEM). **f**, **g** Statistics showing the percentages of CXCR5^+^PD-1^+^ T_FH_ cells (**f**) (IL-12: *P = 0.0105, *P = 0.0107; IL-23: **P = 0.0063, **P = 0.0087; TGF-β: *P = 0.0187,*P = 0.0161; IL-12+TGF-β: **P = 0.0063,**P = 0.0052; IL-23+TGF-β: *P = 0.0452,*P = 0.0211; IL-12+IL-23+TGF-β: *P = 0.0206,**P = 0.0023) and IL-21^+^ cells (**g**) (IL-12: **P = 0.0068, **P = 0.0071; IL-23: **P < 0.0001, **P = 0.0012; TGF-β: *P = 0.0287,**P = 0.0096; IL-12+TGF-β: **P = 0.0023,*P = 0.0129; IL-23+TGF-β: *P = 0.0145,**P = 0.0044; IL-12+IL-23+TGF-β: **P = 0.0084,**P = 0.0082) differentiated from naïve CD4^+^ T cells with anti-CD3/CD28 stimulation and indicated cytokine combinations for 4 days (dots, *n* = 5; bars, mean ± SEM). Data from individuals (dots) and the median values (bars) are shown for (**a**); Mann–Whitney test was used for analysis (**a**). Percentages and numbers are shown for (**b**); Chi-Square test was used for analysis (**b**). Real-time PCR and FACS statistics are shown for individuals (dots, *n* = 3) and mean ± SEM (bars) values, and analysed by Mann–Whitney U-test (**a**, **c**), one-way ANOVA Dunnett’s multiple comparisons test (**d**–**g**). *P < 0.05, **P < 0.01. Results are representative of three independent experiments.

Previous studies including ours revealed that the T_FH_ activity is critical for the effectiveness of human vaccination^12–14,16^. We next asked whether leptin levels affected the T_FH_ generation after vaccination. Among the three cohorts above, peripheral blood mononuclear cells (PBMCs) were collected and stored for the middle-age cohort (18–60 years old, *n* = 76) immunised with influenza vaccines (Fig. 1a, b). When the T_FH_ generation in PBMCs was profiled, we found that the vaccine-induced T_FH_ differentiation correlated with the production of protective antibodies (Supplementary Fig. 1), confirming previous reports that the T_FH_ activity controlled antibody production after vaccination^12,14,16,17^. Importantly, those with high T_FH_ activities (T_FH_ fold-change >1) had significantly higher serum leptin than those with low T_FH_ activities: median, 11.1 vs 6.3 ng/mL (*P* = 0.046, Student’s *t*-test; Supplementary table 4, Fig. 2a). The rate of low T_FH_ activity was 36.8% in the low leptin group, compared to 14.3% in the sufficient leptin group (*P* = 0.031, Chi-Square test; Fig. 2b). These data suggested that leptin might be required for the optimal generation of T_FH_ cells.

To formally test whether leptin potentiates human T_FH_ differentiation, human naïve CD4^+^ T cells were stimulated with anti-CD3/CD28, in the presence or absence of leptin. The addition of leptin enhanced the mRNA of multiple markers for T_FH_ cell differentiation (Fig. 2c). As a control, leptin treatment reduced *IL4* and increased *IL17* transcripts in activated human CD4^+^ T cells (Supplementary Fig. 2a), in agreement with its reported role in suppressing T_H_2 and promoting T_H_17 cells^26–28^. Flow cytometric analysis indicated that leptin potently promoted the expression of CXCR5 (Fig. 2d) and the production of IL-21 (Fig. 2e) from cultured human CD4^+^ T cells in a dose-dependent manner. TGF-β plus IL-12 or IL-23 were shown to promote CXCR5 expression and T_FH_ differentiation^29^. The addition of leptin could also synergise with each of these reported T_FH_-inducing cytokines to enhance the expression of CXCR5 and IL-21 (Fig. 2f, g). The direct effect of leptin in promoting human T_FH_ differentiation supports the notion that low levels of leptin might impair the activities of T_FH_ cells and confers the risk of poor vaccine responses.

### Defective leptin signalling impairs antibody responses in mice

Can leptin regulate antibody responses in vivo? We first detected leptin by immunofluorescent staining and found it was abundantly produced in white pulps including T-cell zones and B-cell follicles (Supplementary Fig. 3a, b), suggesting that CD4^+^ T cells and B cells could access leptin throughout antibody responses. The expression of LepR was higher on T_FH_ cells than on naïve CD4^+^ T cells (Supplementary Fig. 3c) and was further induced by antigen, co-stimulatory signals and leptin (Supplementary Fig. 3d-f).

**Fig. 3:**
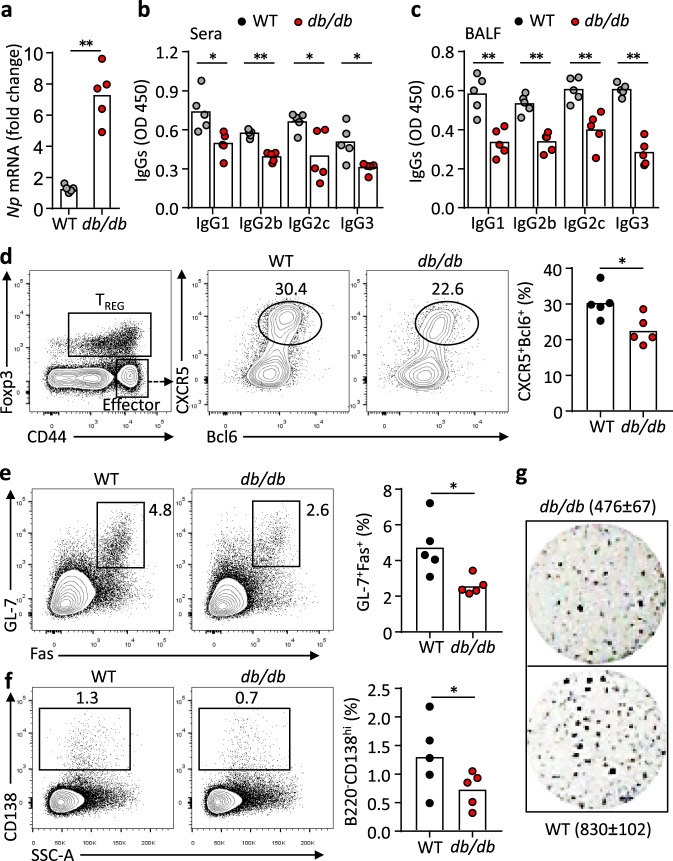
LepR deficiency leads to impaired antibody responses after influenza virus infection. **a** Real-time PCR analysis of nucleoprotein (*Np*) in lung tissues of WT (grey) and *db/db* (red) mice 9 days post H1N1 influenza viral infection (**P = 0.0007). **b**, **c** ELISA measurement of H1N1-specific IgG1, IgG2b, IgG2c and IgG3 in sera (**b**) (IgG1: *P = 0.0190, IgG2b: **P < 0.0001, IgG2c: *P = 0.0299, IgG3: *P = 0.0209) and BALF (**c**) (IgG1: **P = 0.0080, IgG2b: **P = 0.0036, IgG2c: **P = 0.0079, IgG3: **P = 0.0007). **d**–**f** Representative FACS plots and statistics showing CXCR5^+^Bcl6^+^ T_FH_ cells (**d**) (*P = 0.0407), GL-7^+^Fas^+^ germinal centre (GC) B (**e**) (*P = 0.0240) and CD138^high^ antibody-secreting cells (ASCs) (**f**) (*P = 0.0411) in mediastinal lymph nodes. **g** ELISPOT assay showing virus-specific ASCs in spleens. Data are shown for individuals (dots, *n* = 5 per genotype) and mean (bars) values, and analysed by Mann–Whitney U-test (**a**–**f**). *P < 0.05, **P < 0.01. Results are representative of three independent experiments.

To test whether leptin signalling is required to mount normal T_FH_ and humoral responses in vivo, we challenged WT and leptin receptor (LepR)-deficient (*db/db*) mice with H1N1 influenza virus. Compared to WT mice, *db/db* mice showed defective viral clearance (Fig. 3a, Supplementary Fig. 4a). Despite the increased viral antigens, *db/db* mice produced significantly less virus-specific antibodies in sera and bronchoalveolar lavage fluid (BALF) (Fig. 3b, c). Draining lymph node is important for memory T_FH_ formation and vaccination efficacy^30^, flow cytometric analysis revealed a significant reduction of T_FH_ cells (Fig. 3d), accompanied by the decrease in germinal centre (GC) B cells and antibody-secreting cells (ASCs) (Fig. 3e, f) in the mediastinal lymph nodes (mLNs). An Enzyme-Linked ImmunoSpot (ELISPOT) assay also showed a two-fold reduction of ASCs in spleens of *db/db* mice, compared to WT mice (Fig. 3g). We previously showed that inflammatory cytokines induced by acute malaria infection could suppress T_FH_ differentiation^31^. To rule out the possibility that the weakened T_FH_ generation and humoral response in *db/db* mice was caused by the persistence of high virus titres and enhanced inflammatory cytokines, we immunised WT and *db/db* mice with 2,4,6,-trinitrophenyl (TNP)-keyhole limpet hemocyanin (KLH) in complete Freund’s adjuvant (CFA) and we again observed defective T_FH_ and GC B-cell differentiation and reduced antibody production, compared to WT mice (Supplementary Fig. 4b-d). IL-21 is the key cytokine produced by T_FH_ cells that underpins the B-helper function^32–35^. Compared to WT cells, *db/db* CD4^+^ T cells produced less IL-21 (Supplementary Fig. 4e). These data indicate that LepR deficiency impairs T_FH_ and B-cell responses following infection and protein immunisation.

### T-cell-specific LepR is required for optimal T_FH_ and antibody responses

We previously reported that leptin promotes B-cell homeostasis by inhibiting apoptosis and inducing cell-cycle entry^36^. To investigate the role of cell-autonomous leptin signalling in T and B-cell differentiation, we transferred combined T cells and B cells from either WT or *db/db* mice into *Rag1*^*-/-*^ recipient mice. After 1 week, mice were immunised with 4-hydroxy-3-nitrophenyl (NP)-ovalbumin (OVA) in CFA and analysed after 9 days (Supplementary Fig. 5a). The lack of leptin signalling in either T or B cells was sufficient to impair the generation of GC and ASCs (Supplementary Fig. 5b). This observation was also supported by testing the capability between WT and *db/db* T_FH_ cells in supporting GC B cells to produce antibodies in the in vitro co-culture (Supplementary Fig. 5c). Isolated *db/db* T_FH_ cells were less capable than their WT counterparts in this assay while co-culturing *db/db* T_FH_ and *db/db* GC B cells hardly produced antibodies (Supplementary Fig. 5d). To test cell-autonomous leptin signalling in a physiological manner, we reconstituted irradiated *Rag1*^*-/-*^ mice with 50:50 mixed bone marrow (BM) cells from *db/db* or WT (both CD45.2) mice and congenically marked WT (CD45.1) mice. After reconstitution, BM chimeric mice were immunised with NP-OVA in CFA and analysed 9 days after immunisation. An analysis of CD4^+^ T cells showed that *db/db* and WT-derived Foxp3^+^ T_REG_ and total Foxp3^-^CD44^high^ effector subsets were comparable. However, the CXCR5^+^Bcl6^+^ T_FH_ subset was reduced by 2-fold in *db/db* cells, compared to WT cells (Supplementary Fig. 6a), suggesting an intrinsic defect of *db/db* T_FH_ cells. We observed that *db/db*-derived NP^+^IgG1^+^ antigen-specific B cells and CD138^high^ ASCs were reduced compared to counterpart WT cells (Supplementary Fig. 6b). The frequencies of the memory and GC B-cell subsets in NP^+^IgG1^+^ antigen-specific B cells were comparable between *db/db* and WT cells (Supplementary Fig. 6b). These sets of experiments demonstrated that leptin signalling in both T and B cells are required for optimal humoral responses, especially for T_FH_ differentiation and the generation of ASCs.

**Fig. 4:**
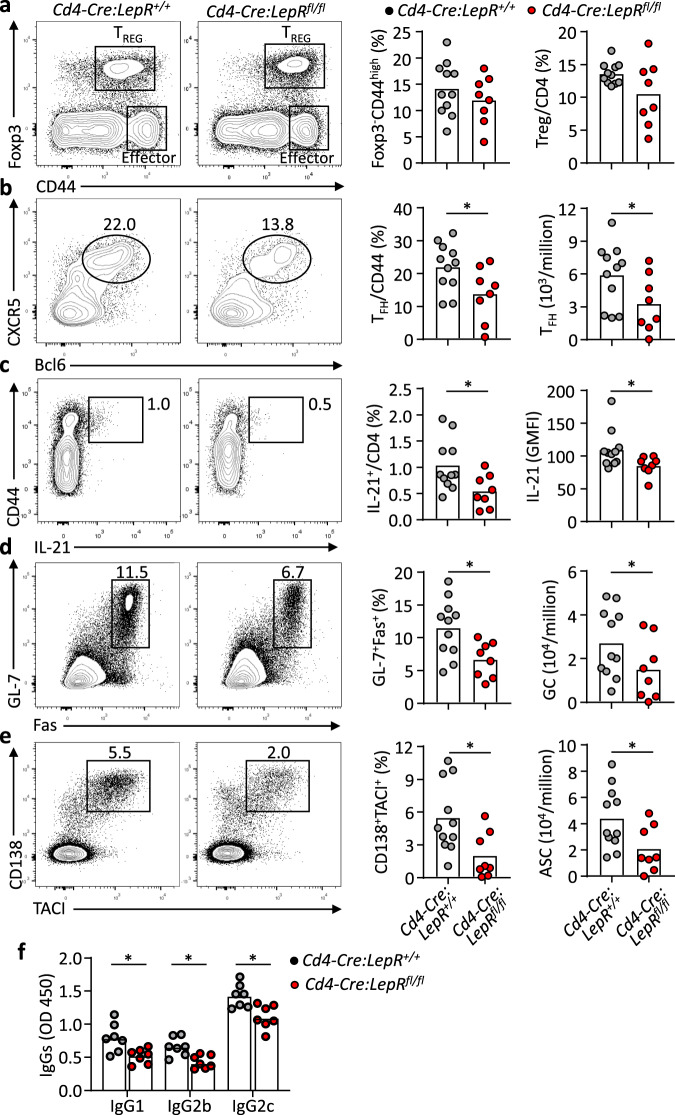
Defective T_FH_ cells responses in *Cd4-Cre:LepR*^*fl/fl*^ mice after influenza virus infection. **a**–**e** *Cd4-Cre:LepR*^*+/+*^ mice (grey, *n* = 11) and *Cd4-Cre:LepR*^*fl/fl*^ mice (red, *n* = 8) were infected with influenza virus A/X-31 (H3N2). Mediastinal lymph nodes were analysed at day 9 post-infection. Representative FACS plots and statistics showing the CD4^+^Foxp3^-^CD44^high^ effector cells, CD4^+^Foxp3^+^ T_REG_ cells (**a**), CD44^+^CXCR5^+^Bcl6^+^ T_FH_ (**b**), (*P = 0.0499, *P = 0.0347), IL-21 production (**c**), (*P = 0.0313, *P = 0.0142), B220^+^GL-7^+^Fas^+^ GC B cells (**d**) (*P = 0.0151, *P = 0.0249) and B220^-^CD138^+^TACI^+^ ASCs (**e**) (*P = 0.0250, *P = 0.0409) 9 days post influenza viral infection. **f** *Cd4-Cre:LepR*^*+/+*^ and *Cd4-Cre:LepR*^*fl/fl*^ mice were intranasally challenged with H1N1 influenza virus and virus-specific IgG1, IgG2b and IgG2c in sera were measured by ELISA 9 days post-infection (*n* = 7 per genotype) (IgG1: *P = 0.0183, IgG2b: *P < 0.0181, IgG2c: *P = 0.0168). Data are shown for individuals (dots) and mean (bars) values, and analysed by Mann–Whitney U-test (**a**–**f**). *P < 0.05, **P < 0.01. Results are representative of two independent experiments.

To evaluate the contribution of T-cell specific leptin signalling to humoral immunity in vivo, we crossed *Lepr* floxed mice with *Cd4-Cre* transgenic mice to specifically delete LepR signalling in T cells. Mutant (*Cd4-Cre:LepR*^*fl/fl*^) and WT (*Cd4-Cre:LepR*^*+/+*^) mice showed comparable B cells, Foxp3^+^ T_REG_ cells and CD44^+^ effector CD4^+^ T cells^37^. Both groups of mice were infected with the H3N2 influenza virus and analysed after 9 days. There was no significant difference between the two groups for the percentages of total CD44^+^ effector CD4^+^ cells or Foxp3^+^ T_REG_ cells (Fig. 4a). However, the CXCR5^+^Bcl6^+^ T_FH_ percentages in effector cells and their numbers in the draining mediastinal LN were almost halved in mutant mice, compared to WT controls (Fig. 4b). We also observed a significantly reduced production of IL-21 in mutant CD44^+^ effector CD4^+^ cells, compared to WT cells (Fig. 4c). On the contrary, the lack of LepR signalling showed negligible effect on the production of major effector cytokine IFN-γ in this model, neither IL-4 nor IL-17 (Supplementary Fig. 7a-c). We also quantified follicular regulatory T (T_FR_) cells, a subset of T_REG_ cells that localise in B-cell follicles to suppress T_FH_ cell-mediated antibody responses^38,39^. There was a trend for the reduction of T_FR_ cells in the absence of LepR signalling but it didn’t reach significance (Supplementary Fig. 7d). Finally, we examined the key question of whether the LepR deficiency in T cells perturbed GC formation and antibody production. The T-cell-specific deletion of LepR led to about 2-fold reduction of GL-7^+^Fas^+^ GC B cells (Fig. 4d) and almost a 3-fold reduction of CD138^+^TACI^+^ ASCs (Fig. 4e), which was accompanied by the significant decrease of influenza virus-specific IgG1 and IgG2 antibodies (Fig. 4f). Therefore, defective leptin signalling autologously restrained T_FH_ numbers and function, which constituted a limiting factor for antibody responses.

### Leptin promotes T_FH_ function via Stat3 and mTOR signalling pathways

**Fig. 5:**
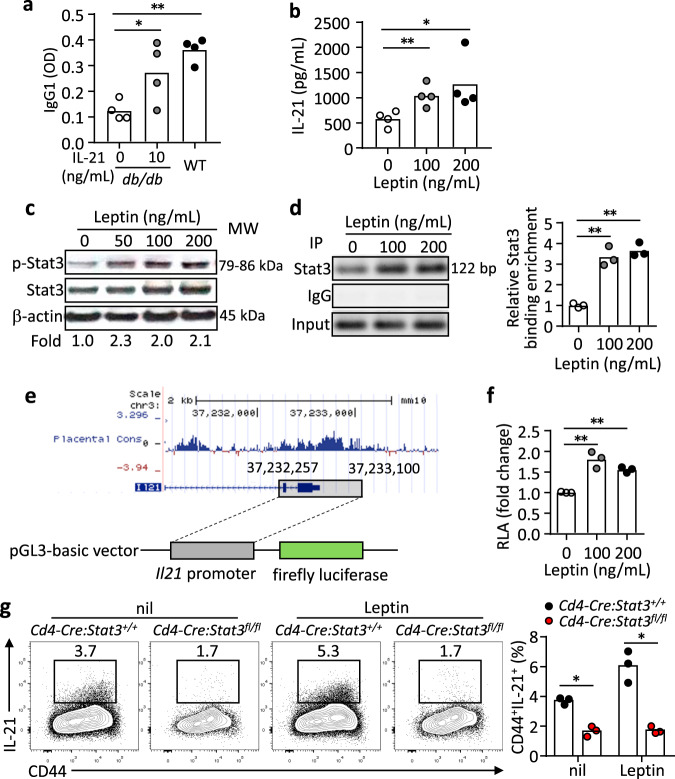
Leptin promotes IL-21 production in a STAT3-dependent manner. **a** B220^-^CD4^+^CD25^-^CD44^+^CD62L^-^CXCR5^+^PD-1^+^ T_FH_ cells (1 × 10^5^) and B220^+^Fas^+^GL-7^+^ GC B cells (1 × 10^5^) from 4-Hydroxy-3-nitrophenylacetyl hapten conjugated to ovalbumin (NP-OVA) immunized WT and *db/db* mice (9 dpi) were co-cultured with 5 µg/mL NP-OVA for 9 days. In the *db/db* T_FH_:WT B-cell culture, IL-21 (10 ng/mL) was added. Anti-NP IgG1 titers in the supernatant were measured by ELISA (IL-21_10 ng/mL: *P = 0.0403, WT: **P = 0.0033). **b** ELISA measurement of IL-21 in cultured naive CD4^+^ T cells from WT mice with anti-CD3/CD28 and leptin (0-200 ng/mL) stimulation for 3 days (100 ng/mL: **P = 0.0100, 200 ng/mL: **P = 0.0441). **c** Western blot showing Stat3 phosphorylation (p-Stat3) in WT naive CD4^+^ T cells stimulated with 200 ng/mL leptin for 2 h. Values showing the fold changes relative to the non-treated control. **d** Binding of Stat3 to the *Il21* promoter. Stat3-ChIP assays were performed on WT naive CD4^+^ T cells treated with 200 ng/mL leptin for 3 h. Results showing PCR products (left) and values for the fold changes in ChIP enrichment relative to non-treated control (right) (100 ng/mL: **P = 0.0096, 200 ng/mL: **P = 0.0043). **e** Schematic of *Il21* promoter construction and luciferase assay. **f** The transcriptional activity of the *Il21* promoter. Dual-Luciferase reporter assay for the *Il21* promoter in 293 T cells with 200 ng/mL leptin treatment for 12 h (100 ng/mL: **P = 0.0082, 200 ng/mL: **P = 0.0016). **g** *Cd4-Cre*:*Stat3*^*+/+*^ or *Cd4-Cre*:*Stat3*^*fl/fl*^ naive CD4^+^ T cells were stimulated under IL-21-inducing conditions with 200 ng/mL leptin for 3 days. Results for IL-21 secretion showing representative FACS plots (left) and statistics (right) (nil: *P = 0.0111, Leptin: *P = 0.0144). Data are shown for individuals (dots) and mean (bars) values, and analysed by two-way ANOVA (**a**) one-way ANOVA (**b**, **d**, **f**) or Mann–Whitney U-test (**g**). *P < 0.05, **P < 0.01. Results are representative of three independent experiments.

Compared to WT cells, effector CD4^+^ T cells in *db/db* or *Cd4-Cre:LepR*^*fl/fl*^ mice produced less IL-21, the key effector cytokine for T_FH_ function, than their WT counterparts (Supplementary Fig. 4e and Fig. 4c), suggesting a functional impairment of T_FH_ cells in the absence of leptin signalling. To test this, we added IL-21 in the T-B co-culture and found the defective B-helper function of *db/db* T_FH_ cells was improved to the levels similar to that of WT T_FH_ cells (Fig. 5a). We also confirmed that leptin directly promoted the production of IL-21 from stimulated mouse CD4^+^ T cells (Fig. 5b), similar to the effects of promoting IL-21 secretion by cultured human CD4^+^ T cells (Fig. 2e).

We next set to identify the molecular mechanism by which leptin stimulates IL-21 production. As a key downstream signal pathway that mediates the function of leptin^22,40^, Stat3 signalling is critical for the generation of both human and mouse T_FH_ cells and IL-21 production by these cells^1,4^. Leptin induced Stat3 phosphorylation in mouse CD4^+^ T cells (Fig. 5c, raw gel blots in Source Data). Using the Chromatin Immunoprecipitation (ChIP) assay, we found that leptin increased the binding of Stat3 to the *Il21* promoter in mouse CD4^+^ T cells (Fig. 5d, raw gel blots in Source Data), suggesting that the leptin-Stat3 axis enhances the *Il21* transcription. To directly examine it, the *Il21* promoter was cloned into a luciferase reporter construct (Fig. 5e), which was then transduced into 293 T cells. Leptin treatment was able to enhance the transcriptional activity of the *Il21* promoter, measured by the luciferase activities (Fig. 5f). In line with this, Stat3-deficient CD4^+^ T cells failed to respond to leptin-stimulated IL-21 production (Fig. 5g). Together, these data suggest that Stat3 mediates leptin-stimulated IL-21 production.

**Fig. 6:**
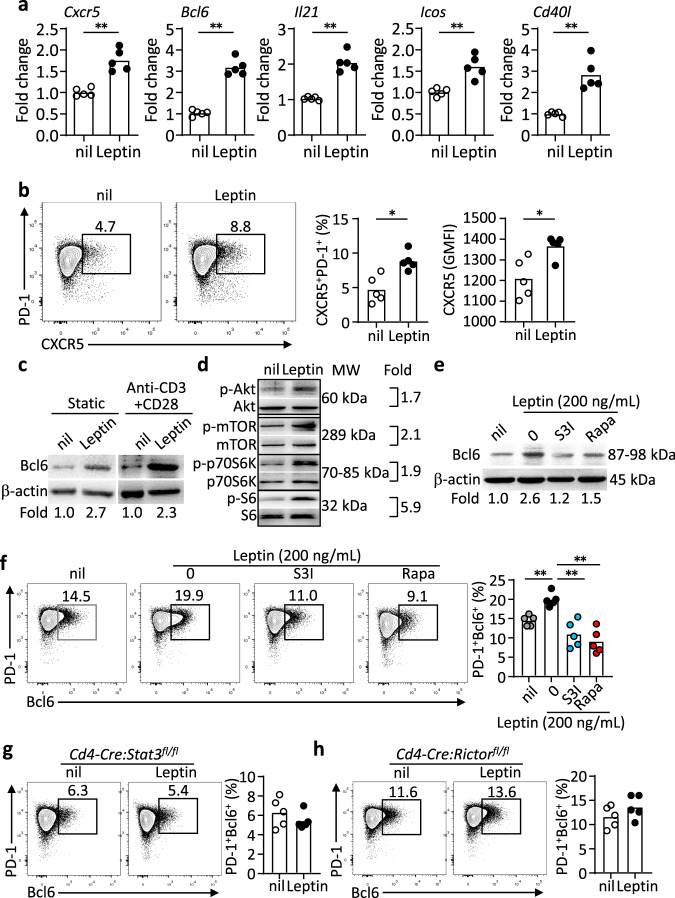
Stat3 and mTOR are dispensable for leptin-induced T_FH_ differentiation and Bcl6 expression. **a** Real-time PCR analysis of mouse T_FH_-related genes in naïve CD4^+^ T cells from WT mice stimulated with anti-CD3/CD28 for 2 days, further treated with leptin for 12 h (*Cxcr5*: **P = 0.0029, *Bcl6*: **P = 0.0004, *Il21*: **P = 0.0007, *Icos*: **P = 0.0028, *Cd40l*: **P = 0.0023) (*n* = 5). **b** Representative FACS plots and statistics of CXCR5^+^PD-1^+^ T_FH_ cells in cultured naïve CD4^+^ T cells from WT mice with anti-CD3/CD28 stimulation for 48 h, followed with or without leptin treatment for 12 h (Ratio: *P = 0.0200, GMFI: *P = 0.0243). **c**–**e** Western blot assay of Bcl6 with or without anti-CD3/CD28 activation (**c**), the phosphorylation of Akt, mTOR, p70S6K and S6 (**d**) and Bcl6 expressions in WT naïve CD4^+^ T cells with Stat3 inhibition (S3I-201, 100 μM), mTOR inhibition (Rapamycin, 200 nM) plus leptin (200 ng/mL) treatment for 12 h (**e**). Values showing the fold changes relative to the non-treated control. **f** Representative FACS plots and statistics of PD-1^+^Bcl6^+^ T_FH_ cells in cultured naïve CD4^+^ T cells from WT mice with anti-CD3/CD28 activation for 2 days, followed with Stat3 inhibition (S3I-201, 100 μM), mTOR inhibition (Rapamycin, 200 nM) plus leptin (200 ng/mL) treatment for 12 h (0 vs nil: **P = 0.0004, S3I vs 0: **P = 0.0023, Rapa vs 0: **P = 0.0026) (*n* = 5). **g**, **h** Representative FACS plots of PD-1^+^Bcl6^+^ T_FH_ cells in cultured naïve CD4^+^ T cells from *Cd4-Cre:Stat3*^*fl/fl*^ mice (**g**) and *Cd4-Cre*:*Rictor*^*fl/fl*^ mice (**h**) with anti-CD3/CD28 activation for 2 days, followed with or without leptin (200 ng/mL) treatment for 12 h (*n* = 5). Data are shown for individuals (dots) and mean (bars) values, and analysed by Mann–Whitney U-test (**a**, **b**), or two-way ANOVA (**f**). *P < 0.05, **P < 0.01. Results are representative of three independent experiments.

In addition to *Il21*, adding leptin in cultured CD4^+^ T cells also increased the mRNA of other major markers for mouse T_FH_ cell differentiation, including *Cxcr5*, *Bcl6*, *Icos* and *Cd40l*, but not *Il4* (Fig. 6a and Supplementary Fig. 2b), suggesting that leptin alone induces the overall T_FH_ differentiation program, consistently with the promoting function of leptin for human T_FH_ differentiation (Fig. 2d). Indeed, not only did leptin treatment enhance the expression of CXCR5 on activated CD4^+^ T cells (Fig. 6b), it also induced the expression of the key T_FH_ transcription factor Bcl6 (Fig. 6c, raw gel blots in Source Data). Both mTOR and Stat3 pathways are required for mouse T_FH_ cell differentiation and function^1,41,42^. Besides the Stat3 pathway (Fig. 5c), lepR signalling also activates the mTOR pathway in cultured CD4^+^ T cells, as measured by the phosphorylation of p70^S6K^ and S6, one major pathway downstream of mTOR (Fig. 6d, raw gel blots in Source Data). We found that leptin-induced Bcl6 expression was blunted by the treatment of the inhibitors for the Stat3 pathway (S3I-201) or the mTOR pathway (Rapamycin) (Fig. 6e, f, raw gel blots in Source Data). It has been shown that mTOR kinase complexes 1 and 2 (mTORC1 and mTORC2) are both required for T_FH_ responses, with mTORC2 more specifically regulating T_FH_ differentiation and mTORC1 mainly promoting CD4^+^ T-cell proliferation^41–43^. Correspondingly, CD4^+^ T cells from *Cd4-Cre:Stat3*^*fl/fl*^ or *Cd4-Cre:Rictor*^*fl/fl*^ mice were unable to upregulate Bcl6 expression upon the leptin treatment (Fig. 6g, h). Collectively, leptin induces the activation of Stat3 and mTOR pathways to promote T_FH_ differentiation and function.

### Leptin insufficiency impairs vaccination-mediated protection

After establishing that the genetic ablation of leptin signalling impaired humoral immunity, we next asked whether the physiological restriction of leptin production attenuates antibody responses in vivo. We first employed an acute fasting model that had been shown to reduce leptin production^25^. OT-II CD4^+^T cells that specifically recognise the OVA peptide were adoptively transferred into congenic WT mice that were subsequently immunised with NP-OVA in CFA. Mice were fed with a normal diet (Fed) or acutely fasted (Fast) on day 7 and 8 when T_FH_ and GC responses were at peak^7^. Fasted mice were divided into two groups, which were treated with either leptin to compensate for fasting-induced leptin insufficiency or PBS as control (Supplementary Fig. 8a), to delineate the effect of leptin insufficiency on the antibody response. The expansion of transferred CD45.1^+^ OT-II cells in recipient mice with acute fasting was significantly inhibited (Supplementary Fig. 8a, b) while the differentiation of CXCR5^+^Bcl6^+^ T_FH_ cells was further repressed for more than 2-fold (Supplementary Fig. 8a,b). Leptin supplementation during acute fasting partially improved the expansion of OT-II cells and largely rescued the T_FH_ differentiation (Supplementary Fig. 8a, b). Importantly, leptin treatment completely restored serum IL-21 levels, which was reduced by over 2-fold by acute fasting for 48 h (Supplementary Fig. 8c). These results demonstrate that the physiological restriction of leptin by acute fasting diminished both the generation and function of T_FH_ cells. We also measured the generation of NP-specific Fas^+^GL-7^+^ GC B cells and CD138^+^ ASCs (Supplementary Fig. 8d). Although leptin supplementation was unable to rectify the generation of NP-specific B cells weakened by acute fasting (Supplementary Fig. 8e), it fully recovered GC and ASC differentiation (Supplementary Fig. 8d,e). Therefore, leptin insufficiency caused by acute fasting plays a central in suppressing T_FH_ function and humoral immunity.

**Fig. 7:**
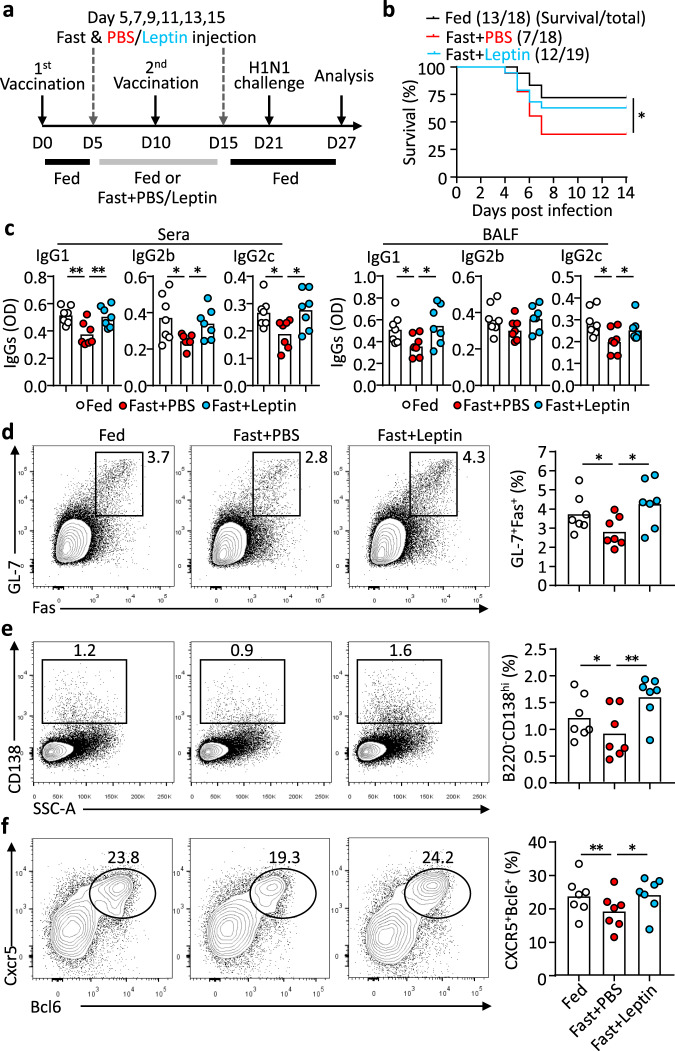
Fasting impairs vaccination-mediated protection, which can be compensated by leptin replacement. **a** WT female mice were immunized (s.c.) with human influenza vaccine, following various feeding regimens: normal diet (Fed) (*n* = 18, black line), fasting with PBS (Fast + PBS) (*n* = 18, red line), fasting with leptin treatment (Fast + leptin) (*n* = 19, cyan line); mice were intranasally challenged with H1N1 influenza virus and analysed 6 days post-infection. **b** Accumulative survival curve of mice after viral challenge (Fast + PBS vs Fed: *P = 0.0369). **c** ELISA measurement of H1N1-specific IgG1, IgG2b and IgG2c in sera (IgG1: **P = 0.0073, **P = 0.0076; IgG2b: *P = 0.0133, *P = 0.0258; IgG2c: *P = 0.0326, *P = 0.0252) and bronchoalveolar lavage fluid (BALF) (IgG1: *P = 0.0488, *P = 0.0240; IgG2c: *P = 0.0271, *P = 0.0481). **d**–**f** Representative FACS plots and statistics showing frequencies of GC B cells in B cells (**d**) (*P = 0.0446, *P = 0.0197) and ASCs in lymphocytes (**e**) (*P = 0.0261, **P = 0.0081), and T_FH_ cells in CD44^+^Foxp3^+^ CD4^+^ T cells (**f**) (**P = 0.0022, *P = 0.0197) in the mediastinal lymph nodes 6 days post-infection. Data are shown for individual (dots, *n* = 7) and mean (bars) values, and analysed by Log-rank tests (**b**), two-way ANOVA (**c**–**f**). *P < 0.05, **P < 0.01. Results are representative of two independent experiments.

We next turn to address the question of whether leptin insufficiency during vaccination affects vaccination-mediated protection. WT mice were fed with normal diet (Fed) or alternate-day fasted from day 5 to 15 (Fast) following influenza vaccination. Alternate-day fasting immediately reduced the leptin levels and body weight, which could be recovered by normal feeding (Supplementary Fig. 9). The choice of the fasting period from day 5 to 15 was designed not to affect T-cell priming (before day 5) and to selectively target T_FH_ differentiation, which starts around day 5^7^. After a period of normal feeding from day 16 to 21 so that fast mice recovered body weight (Supplementary Fig. 9), mice were challenged with the H1N1 influenza virus at a median lethal dose (LD50). Fasted mice were again divided into two groups, which were treated with either leptin or PBS as the control on alternate fasting days from day 5 to 15 (Fig. 7a). Compared to normal feeding, alternate-day fasting during the period of vaccination greatly reduced the survival rate of virus-infected mice from 72.2% to 38.9% (Fig. 7b). Noticeably, leptin replacement on alternate fasting days almost completely restored vaccination-mediated protection in fasted mice, with a survival rate of 63.2% (Fig. 7b). Influenza vaccine-rendered protection is considered predominantly mediated by humoral immunity^44,45^. We, therefore, examined virus-specific antibody production and revealed that the decreased antibody production in alternate-day fasted mice was rescued by leptin replacement (Fig. 7c). Correspondingly, flow cytometric analysis demonstrated that alternate-day fasting retarded the generation of CXCR5^+^Bcl6^+^ T_FH_ cells, as well as GL-7^+^Fas^+^ GC B cells and CD138^high^ ASCs but this was also compensated by leptin treatment (Fig. 7d–f). To further examine whether the observed effect of GC and T_FH_ response by fasting-induced leptinemia and leptin replacement was dependent on leptin signalling in T cells, we conducted a similar experiment in *Cd4-Cre:LepR*^*fl/fl*^ mice. Mice were fed with normal diet or alternate-day fasted from day 5 to 15 following influenza vaccination and then analysed the antibody response at day 21 when bodyweights had recovered (Supplementary Fig. 10a, b). In contrast to wild-type mice, alternate-day fasting or leptin replacement didn’t show noticeable effect on T_FH_ cell differentiation or GC and ASC formation (Supplementary Fig. 10c, d). Intriguingly, fasting or leptin treatment didn’t change the production of vaccine-specific IgG2c and IgG2b antibodies but altered IgG1 antibodies to all three viral strains (Supplementary Fig. 10e). The results from the physiological restriction of leptin support the conclusion that leptin insufficiency is a significant risk factor for T_FH_ function, antibody responses and vaccination-mediated protective humoral immunity.

## Discussion

Metabolism plays a fundamental role in regulating the immune system. Not only are the energetic and biosynthetic pathways within immune cells essential in controlling immune cell development, activation, functional specification and fate determination^18–20^, but the state of systemic metabolism can also directly influence the immune system through signals conveyed by hormones and cytokines, in which the adipokine leptin has been extensively studied^22,23,40^. Leptin specifically regulates the differentiation of T-cell functional subsets. It enhances T_H_1 but suppresses T_H_2 cytokine production^26^. We and others have demonstrated that leptin activates the Stat3 pathway to promote the generation of T_H_17 cells^27,28,37^, while it limits the proliferation of T_REG_ cells through the activity of mTOR pathway^46–48^. Moreover, leptin has been shown to regulate the metabolic fitness of CD8^+^ T cells and control their effector function in the tumour microenvironment and the responses to anti-PD-1 therapies^49–51^. In this study, we revealed leptin in promoting T_FH_ function and supporting antibody responses.

The involvement of leptin in antibody responses was hinted at by previous observations that, compared to WT mice, *db/db* mice produced less protective antibodies to a vaccine against *Helicobacter pylori*^52^, and fewer autoantibodies in the pristine-induced model for lupus^53^. However, the mechanism underlying these observations was unknown. By testing leptin treatment in cultured PBMCs from patients of rheumatoid arthritis, Wang et al. reported the leptin treatment could promote T_FH_ function by enhancing IL-6 production in PBMCs although the study didn’t address whether such regulation was T-cell intrinsic or not^54^. Using a set of experiments, our results identified T_FH_ cells as the effector CD4^+^ T subset that is particularly affected by the lack of leptin signalling. We found that leptin signalling was not only required for optimal T_FH_ cell differentiation but was also sufficient to promote T_FH_ cell generation and directly induce IL-21 production in mice and humans. In culture CD4^+^ T cells, leptin stimulated the *ICOS* expression in human cells while both *Icos* and *Cd40l* in mouse cells, suggesting the function of leptin in broadly enhancing T_FH_ effector function. It remains to be determined whether the regulation of ICOS and CD40L is directly targeted by leptin signalling or in an indirect manner such as through the upregulation of Bcl6. We have previously shown that leptin signalling is required for B-cell homeostasis^36^. In line with this, we also found a cell-autonomous role for leptin in the expansion of antigen-specific B cells and the differentiation of ASCs in the experiment of mixed transfer of T and B cells in RAG-1-deficient mice. Intriguingly, although the effects of alternate-day fasting or leptin replacement on GC and T_FH_ cells were largely dependent on T-cell intrinsic leptin signalling, the production of IgG1 but not IgG2 antibodies was affected by T-cell extrinsic leptin signalling. Future studies are required to dissect leptin function intrinsic to B cells or other cell types in humoral immunity. One caveat of this study is the lack of investigation of how leptin regulates antibody affinity maturation. Reduced T_FH_ and GC responses in the absence of leptin signal suggest leptin is required for optimal GC reaction underlying antibody affinity maturation, which should be formally evaluated in future. In conclusion, the reduction of leptin triggers multiple mechanisms of actions to restrain adaptive immunity, in this case, humoral immunity. We hold the view that leptinemia-associated immune incompetence constitutes a safeguard for energy-saving and sustaining the body’s basic requirements during malnutrition.

Our results demonstrated that the non-responder groups for both influenza and HBV vaccinations were highly enriched in individuals with low serum leptin (Fig. 1). On the other hand, serum leptin levels didn’t always positively correlated with absolute antibody titres post-vaccination or antibody titre changes in adults with influenza or HBV vaccines (Supplementary Fig. 11), in agreement with a previous publication^55^. Altogether, our data support the notion that leptin is a natural regulator of T_FH_ cells in the general population. This should not be interpreted as ‘higher leptin levels, stronger vaccine responses’. Instead, in our opinion, leptin represents a metabolism-mediated threshold factor that is required to mount normal vaccine responses. In other words, a low level of leptin is a profound risk factor for poor vaccine responses. It should also be noted that some individuals with low leptin levels could still generate relatively normal antibody responses, which implies that the negative impact caused by weak leptin signalling might have been compensated by other factors. For example, we previously reported that IL-6, rather than leptin, was highly elevated in patients with rheumatoid arthritis, which correlated with the generation of pathogenic T_FH_ cells in this autoimmune disease^5^. It should be noted that females have higher leptin levels than males when matched by age, weight, or body fat^25^ while females also typically develop higher antibody responses following vaccination than males^56^. Therefore, it will be important to further investigate the role of leptin in sexual dimorphism to humoral immunity and vaccine response.

Understanding individual immune responses to vaccination and identifying related immune signatures or biomarkers are essential for vaccine development^57,58^. Emerging evidence suggests that T_FH_ cells represent a major limiting factor for vaccine efficacy. For example, the induction of functional T_FH_ subsets was shown to correlate with effective antibody responses to influenza vaccination^12,14,17^. In addition, in the most promising HIV vaccine trial to date (ALVAC+ AIDSVAX, RV144), the higher frequency of HIV-specific IL-21^+^ T_FH_ cells was associated with protective antibody responses^16^. Therefore, we and others have proposed to target T_FH_ function as a new approach to improve vaccine efficacy^59,60^. Through association studies using human samples and mechanistic studies using mouse models, we have provided solid evidence to link levels of the metabolic hormone leptin, T_FH_ cell function and vaccine responses. The respective contribution of leptin from systemic production and autocrine secretion from immune cells in the reinforce of vaccine responses remains unclear and needs further investigation. Nevertheless, we suggest that individuals with low levels of leptin should be particularly targeted with strategies to promote their T_FH_ function and enhance vaccine responses. For instance, high-dose and prolonged antigen stimulation favours T_FH_ generation^6^. Indeed, the high-dose influenza vaccine has been shown to elicit greater T_FH_ activation in older adults^61^, which can at least partially explain the increased efficacy of high-dose influenza vaccine in elderly individuals^62^ with reduced leptin sensitivity^63^. Understanding the mechanisms underlying varying vaccine responses among healthy individuals will not only provide critical information on the regulation of T_FH_ function in non-pathogenic conditions but is also required to guide the optimisation of immunisation strategies and the development of new vaccines^44,58,59^. Overall, the identification of leptin as a natural regulator of T_FH_ cell function and vaccine responses has far-reaching implications for vaccine development.

## Methods

### Study cohorts

Two cohorts of influenza vaccination were examined. Elderly adult healthy volunteers (*n* = 83, >65 years old) were recruited for the vaccination of 2008/2009 seasonal influenza vaccine (Seqirus, Australia). Paired sera samples were taken before and 21 days after vaccination. Adult healthy volunteers (*n* = 76, 18–60 years old) were recruited for the vaccination with trivalent or quadrivalent seasonal influenza vaccine (The Cambridge BioResource, Cambridge, UK^24^, Peter Doherty Institute for Infection and Immunity, University of Melbourne, Australia^14^). Paired sera samples were taken before, 7 and 28 or 36 days after vaccination. Young adults (*n* = 22, 18–25 years old) were recruited for the vaccination of Engerix-B® (Hepatitis B surface antigen recombinant (yeast) vaccine, GSK) in Peking University People’s Hospital (Beijing, China). Paired sera samples were taken before and 14 days after vaccination. Detailed demographics were shown in Supplementary tables 5-7. PBMCs from healthy individuals were enrolled to donate blood for this study. Antibody titters were determined by hemagglutination inhibition (HI) assays (cohorts from Seqirus, and the University of Melbourne), Luminex® 100/200™ System (cohorts from The Cambridge BioResource), and Abbott Architect i2000 immunoassay analyser (cohorts from Peking University People’s Hospital). Participants provided written informed consent according to the ethics approved by the Monash University Human Research Ethics Committee (elderly with flu vaccine), Lisbon Academic Medical Center Ethics Committee, reference no. 505/14, (adults with flu vaccine), the University of Melbourne Human Ethics Committee, (ID 1443389.3 and 1443540), the Mercy Health Human Research Ethics Committee (ID R14/25), and the Australian Red Cross Blood Service (ARCBS) Ethics Committee (ID 2015#8). (adults with flu vaccine), and Peking University People’s Hospital Ethics Committee 2015PHB2010-01 (adults with HBV vaccine), and the Ethical Committee of Renji Hospital KY2019-161 (healthy individuals to donate blood) accordingly.

### Mice

Wild-type (WT) C57BL/6, *Rag1*^*−/−*^ (B6.129S7-*Rag1*^*tm1Mom*^/J), leptin receptor-deficient (B6.BKS(D)-*Lepr*^*db*^/J*, db/db*), CD45.1 B6.SJL-*Ptprca Pepcb*, *Cd4-Cre (*B6.Cg-Tg(Cd4-cre)*)*, *Stat3*^*flox*^ (B6.129S1-*Stat3*^*tm1Xyfu*^*/J*), *Lepr*^*flox*^ (B6.129P2-*Lepr*^*tm1Rck*^/J), OT-II (B6.Cg-Tg(TcraTcrb)425Cbn/J) mice were obtained from Jackson Laboratory and *Rictor*^*flox*^ (Rictorf^lox/flox^ (Rictor^tm1.1Klg^/SjmJ)) mice were provided by Dr Lilin Ye (Institute of Immunology, Third Military Medical University (Army Medical University))^43^. Female mice (7–10 weeks of age) on C57BL/6 background were used in this study, and maintained in specific pathogen-free (SPF) animal facilities in the animal facilities of The University of Hong Kong, The Australian National University, Renji Hospital, Shanghai Jiao Tong University School of Medicine. All experiments were performed under the animal welfare guidelines under approved protocols of The University of Hong Kong, The Australian National University and Shanghai Jiao Tong University.

### Influenza virus challenge

H1N1 strain A/Puerto Rico/8/1934 (PR8), or H3N2 strain A/X-31 influenza virus was obtained in the allantoic cavity of 10-day-old embryonated hens’ eggs at 37 °C with 65% humidity for 48 h^64^. The virus was concentrated from the allantoic fluid and purified in a 10–50% sucrose gradient by centrifugation at 25,000 × *g*, 4 °C for 2 h. Virus samples were further inactivated with 0.25% formalin (v/v) at 4 °C for 7 days. The 50% lethal dose (LD_50_) of pdmH1N1 in WT female mice was determined after serial dilution of the viral stock, and LD_50_ dose was used in viral challenge experiments. Female WT and *db/db* mice (7–10 weeks of age), were anaesthetized with isoflurane before being intranasally challenged with 30 μL of pdmH1N1 diluted in PBS. *Cd4-Cre* and *Cd4-Cre:Lepr*^flox^ mice were intranasally challenged with 10^4^ PFU (plaque-forming unit, PFU) A/X-31(H3N2) diluted in 30 μL of PBS after isoflurane anaesthesia.

### Bone marrow chimaeras

*Rag1*^*−/−*^ mice were irradiated and reconstituted with 50:50 mixed bone marrow (BM) cells from *db/db* or WT (both CD45.2) mice and congenically marked WT (CD45.1) mice. After 4–6 weeks of reconstitution, tail bleeding was analysed by FACS to confirm the success reconstitution of the hematopoietic system.

### Vaccination and immunisation

Female WT mice, *Cd4-Cre:LepR*^*fl/fl*^ mice (7–10 weeks of age) were subcutaneously immunized with 100 μL of diluted human flu vaccine (Influsplit Tetra^®^ 2016/2017 GSK) containing 1 µg of HA derived from the H1N1 influenza virus strain A/California/7/2009(H1N1)pdm09 at the left and right flanks. A booster immunization was performed 10 days later.

Female WT, *db/db*, CD45.2 recipient mice transferred with CD45.1^+^OT-II cells, WT and *db/db* BM reconstituted *Rag1*^*−/−*^ chimaeras, and *Rag1*^*−/−*^ mice transferred with mixed naïve CD4^+^ T:B cells bone marrow, were subcutaneously immunized with 100 μg of NP-OVA or TNP-KLH emulsified in Complete Freund’s Adjuvant (CFA).

### Fasting and leptin administration

Vaccinated mice with human flu vaccine were divided into three groups on day 5: normal diet (Fed) and alternate-day fasting with i.p. injection of 200 μL of PBS (Fast + PBS) or leptin (Fast + Leptin) (1 μg/g per mouse initial body weight) twice daily at 9 am and 6 pm every other day from day 5 to 15. Other days all mice were switched to the normal diet.

CD45.2 recipient mice with CD45.1^+^OT-II cells transfer and NP-OVA/CFA immunisation were fed with normal diet (Fed), fasting with PBS (Fast + PBS) or leptin (Fast + Leptin) from day 7 to day 8.

### Cell transfer

1 × 10^5^ PI^-^B220^-^CD4^+^CD25^-^CD44^-^CD62L^+^ naïve CD4^+^ T cells from CD45.1^+^OT-II mice were FACS sorted and intravenously transferred to *Rag1*^*−/−*^ mice. 3 × 10^6^ PI^-^B220^-^CD4^+^CD25^−^CD44^−^CD62L^+^ naïve CD4^+^ T cells and 6 × 10^6^ PI^-^B220^+^CD3^−^ cells were FACS sorted from WT and *db/db* mice were mixed and intravenously transferred to *Rag1*^*−/−*^ mice.

### ELISPOT

H1N1-virus-specific antibody-secreting cells (ASCs) in the spleens of H1N1-challenged WT and *db/db* mice were identified by ELISPOT^64^. Briefly, 96-well plates (Cat. # MSIPS4W10, Millipore) were pre-coated with purified H1N1 influenza virus A/PR/8/34 (5 mg/mL) at 4 °C overnight. Plates were washed and blocked with RPMI 1640 with 10% FBS at room temperature for 1 h. 1 × 10^4^ PI^-^CD3^-^B220^+^ cells from spleens of WT and *db/db* mice with H1N1 influenza virus on 9 days post-challenge were sorted and cultured for 12 h. Goat Anti-Mouse IgG Alkaline Phosphatase was then added after thoroughly wash and incubated for 1 h at room temperature. 5-Bromo-4-Chloro-3-Indolyl Phosphate (BCIP)/Nitro Blue Tetrazolium (NBT) substrate solution (Sigma–Aldrich) was added. Spots were monitored, stopped by gently washing, identified and counted by the Photoshop CS4 software (Adobe).

### ELISA

TNP-, NP-specific antibodies in serum of TNP-KLH, NP-OVA immunized mice, H1N1- specific IgG1, IgG2c, IgG2b and IgG3 in serum and BALF of virus-challenged mice were measured with sandwich ELISA. Biotin anti-mouse IgG1 antibody (RMG1-1, 1:500 dilution), biotin anti-mouse IgG2c (RMG2a-62, 1:500 dilution), biotin anti-mouse IgG2b (RMG2b-1, 1:500 dilution), biotin anti-mouse IgG3 (RMG3-1, 1:250 dilution) and HRP streptavidin were purchased from Biolegend. TMB substrate was added and the optical density (OD) values were obtained with a microplate reader (SpectraMax 190, Molecular Devices). IL-21 levels in the supernatant of cultured mouse naive CD4^+^ T cells and human naive CD4^+^ T cells were measured using ELISA kits (Mouse IL-21 ELISA Ready-SET-Go!™ Kit, Cat. # BMS6021) and (Human IL-21 ELISA Ready-SET-Go! Kit, Cat. # BMS2043) with the manufacturer’s instructions (eBioscience), respectively. Mouse serum leptin levels, and human serum leptin levels were measured using the Mouse/Rat Leptin Quantikine ELISA Kit (Cat. # SMOB00B, R&D Systems), and Human Leptin Quantikine ELISA Kit (Cat. # SLP00, R&D Systems), respectively.

### Hemagglutination inhibition assays

Antibody titres in the sera were determined by hemagglutination inhibition assays with previously reported protocol^13^. Sera samples were pre-treated with a receptor-destroying enzyme (RDE) for 18 h at 37 °C in a water bath and then heated for 1 h at 56 °C. Sera were 2-fold diluted with saline solution (0.9%) in 96-well plates. Four agglutinating doses of A/H1N1, A/H3N2, or B strain were added to each well, mixed with sera samples and incubated at room temperature for 1 h. Fresh turkey red blood cells were diluted and added to each well, after 1-h incubation at room temperature, the presence of agglutination inhibition was evaluated. Antibody titres are expressed as the inverse sera dilution that inhibited viral agglutination. The fold changes of serum HI titres of H1N1, H3N2 and B strains on day 7, day 42 were calculated by dividing values at day 0^12,14^.

### Flow cytometry

Single-cell suspensions from spleens and draining lymph nodes were prepared and stained with fluorochrome-conjugated monoclonal antibodies: anti-CD45.2-APC (104), anti-CD45.1 BV510 (A20), anti-B220-AF700 (RA3-6B2), anti-GL-7-FITC (GL7), anti-Fas-PE-Cy7 (SA367H8), anti-CD138-BV711 (281-2), anti-IgD APC/Cy7 (11-26 c.2a), anti-CD8-AF700 (53-6.7), anti-CD44-PE/Cy7 (IM7), anti-CD62L-BV711 (MEL-14), anti-TACI-PE (8F10), anti-PD-1-BV421 (29 F.1A12), anti-IgG1-APC (RMG1-1), anti-IL-17A-FITC (TC11-18H10.1), anti-IFN-γ-PE-Cy7 (XMG1.2), anti-IL-4-PE/Dazzle 594 (11B11), anti-CD19-FITC (6D5), biotin anti-mouse CD185 (L138D7) mice antibodies, anti-CCR7-APC/Cy7 (G043H7), anti-CD45RA-PE-Cy7 (HI100), anti-CXCR5-APC (J252D4), anti-PD-1-BV711 (EH12.2H7) human antibodies, and Alexa Fluor 647 Streptavidin were purchased from Biolegend. Anti-IgM-BV421 (R6-60.2), anti-CD38-FITC (90/CD38), anti-Bcl6-PE (K112-91), anti-CD4-BV510 (GK1.5) were purchased from BD Pharmingen. Anti-IL-21-PE (mhalx21), anti-Foxp3-FITC (FJK-16s), anti-human IL-21-PE (eBio3A3-N2 (3A3-N2)) were purchased from eBioscience. Streptavidin-PE-Cy5.5 was obtained from Thermo Fisher. Leptin receptor (LepR) (R&D) with secondary antibody Alexa Fluor 488 Donkey Anti-Goat IgG H&L (Abcam). NP-BSA-Biotin was obtained from Biosearch Technologies. For intracellular cytokine staining, cells were stimulated with PMA (Sigma–Aldrich), Ionomycin (Sigma–Aldrich), Monensin (Biolegend), or Brefeldin A (BD Pharmingen) for 5 h and stained with the Fixation/Permeabilization Solution Kit (Cat. # 554714, BD Pharmingen). Intracellular staining of Bcl6 and Foxp3 was performed using the Foxp3/Transcription factor staining set (Cat. # 00-5523-00, eBioscience). 7-AAD (7-Aminoactinomycin D) (Thermo Fisher), PI (Propidium Iodide) (Biolegend) and Zombie Aqua™ Fixable Viability Kit (Cat. # 423102, Biolegend) were used to distinguish live/dead cells.

### Cell culture

Human T_FH_ cell culture. Human PI^-^CD4^+^CD45RA^+^CCR7^+^ naive cells were sorted (FACSAria III, BD) and cultured in anti-CD3 (5 μg/mL) and anti-CD28 (1 μg/mL) coated 96-well plates, with cytokines: IL-12 (1 ng/mL, PeproTech), IL-23 (20 ng/mL, PeproTech), TGF-β (1 ng/mL, PeproTech), and combinations of indicated leptin (0–800 ng/mL, PeproTech) for 4 days.

Mouse T_FH_ cell culture. Naive CD4^+^ T cells from WT, *Cd4-Cre*, *Cd4-Cre*:*Stat3*^*fl/fl*^ were sorted (FACSAria III, BD) and cultured in anti-CD3 (1 μg/mL) and anti-CD28 (1 μg/mL) coated 96-well plates with 0-200 ng/mL leptin treatment for 3 days. For the intracellular staining of Bcl6 expression, naive CD4^+^ T cells from *Cd4-Cre*, *Cd4-Cre:Stat3*^*fl/fl*^ and *Cd4-Cre:Rictor*^*fl/fl*^ mice were stimulated with anti-CD3 (1 μg/mL, Biolegend) and anti-CD28 (1 μg/mL, Biolegend) for 2 days, further with Stat3 inhibition (S3I-201,100 μM, Selleck Chemicals), mTOR inhibition (Rapamycin, 200 nM, Selleck Chemicals), with or without leptin (200 ng/mL) treatment for 12 h.

Mouse T_FH_:B cells co-culture. B220^-^CD4^+^CD25^-^CD44^+^CD62L^-^CXCR5^+^PD-1^+^ T_FH_ cells and B220^+^Fas^+^GL-7^+^ GC B from NP-OVA immunized WT and *db/db* mice were sorted by FACS (purity > 95%). 1 × 10^5^ T_FH_ cells and 1 × 10^5^ GC B cells were co-cultured with 5 µg/mL NP-OVA for 9 days. In the *db/db* T_FH_:WT B-cell culture, IL-21 (10 ng/mL, PeproTech) was added and with a half-volume of medium change every 3 days.

### Western blot

Cultured naïve CD4^+^ T cells were lysed with RIPA buffer (Sigma–Aldrich) with protease inhibitor cocktail (Roche Diagnostics). Primary antibodies used for immunoblotting were as follows: anti-Akt, anti-phospho-Akt (Thr308), anti-Stat3, anti-phospho-Stat3 (Tyr705), anti-mTOR, anti-phospho-mTOR (Ser2448), anti-p70S6K, anti-phospho-p70S6K (Thr389), anti-S6, anti-phospho-S6 (Ser240/244) and anti-β-actin were purchased from Cell Signalling Technology. Anti-Bcl6 (clone: IG191E/A8) were obtained from Biolegend. Followed by horseradish peroxidase-conjugated (HRP) conjugated secondary antibodies (Anti-rabbit IgG), and detected by chemiluminescent substrates (GE Healthcare Biosciences). Gel bands were analysed with Photoshop CS4 software (Adobe), and fold changes were analysed with ImageJ software (NIH).

### Immunofluorescence staining and confocal microscopy

LepR expression on naïve CD4^+^ T cells and T_FH_ cells from WT mice were stained with leptin receptor (LepR) (Polyclone, R&D) with secondary antibody Alexa Fluor 488 Donkey Anti-Goat IgG H&L(Abcam).

Leptin-secreting cells in the spleen of WT mice were stained with the following antibodies conjugated with fluorochromes: Alexa Fluor 488 anti-mouse IgM (Clone: RMM-1, Biolegend), Brilliant Violet 421 anti-mouse CD4 (Clone: GK1.5, Biolegend), Alexa Fluor 555 leptin (Polyclone, Bioss), Brilliant Violet 421 anti-mouse CD19 (Clone: 6D5, Biolegend), Alexa Fluor 488 anti-mouse F4/80 (Clone: BM8, Biolegend), Nuclei were identified by DAPI staining. Images were obtained with confocal microscopy (Zeiss LSM 710, Zeiss), and analysed with Photoshop CS4 software (Adobe) and Zen software (Zeiss).

### Chromatin immunoprecipitation

Chromatin immunoprecipitation (ChIP) was performed using the EZ-ChIP™-Chromatin Immunoprecipitation Kit (Cat. # 17-371, Millipore) following the manufacturer’s instructions. Briefly, 2 × 10^6^ WT naive CD4^+^ T cells were treated with leptin (0-200 ng/mL) for 3 h, cells were then fixed with formaldehyde, sonicated to shear DNA to lengths between 200 and 1000 base pairs. Immunoprecipitation was performed using anti-Stat3 antibody (Cell Signalling Technology) and DNA was purified and analyzed by quantitative real-time PCR (ABI 7900) using primers targeting the *Il21* promoter as shown: sense 5’-TGCCGCTGCTTTACTCATTG-3’ and antisense 5’-GCACCGTCAGCTTTCAGAGA-3’^65^ (Supplementary table 8).

### Luciferase reporter assay

The 843-bp region (chr3:37232257-37233100) of IL21 promoter was amplified with the sense 5’-CTTGGTACCAAAAAGCATAGTCATCACCC-3’ and antisense 5’-GCCGGTACCGATCTTACCTTTACA-3’ containing kpnI digestion site (Supplementary table 8). PCR product was digested with kpnI and ligated with kpnI digested PGL-3-basic. After ligation and cloning, the plasmid was extracted and validated by Sanger sequencing. pGL-3-IL21 plasmid was transfected into 293 T cells using lipofectamine 2000 with the manufacturer’s protocol. After 6 h of transfection, cells were treated with leptin for 24 h, and finally treated with PMA (50 ng/mL) + Ionomycin (1 μg/mL) for 12 h. Cells were collected, lysed and luciferase activity was measured with Dual-Glo® Luciferase Assay System (Cat. # E2920, Promega) in a Victor X3 Multilabel reader (PerkinElmer).

### Quantitative real-time PCR

Total mRNA was extracted using TRIzol reagents (Invitrogen), and cDNA was synthesized with cDNA Synthesis Kits (Cat. # RR036A, Takara). The mRNA level of the target gene was measured by real-time PCR (ABI 7900 System) using the SYBR Green Master Mix (Cat. # 4385612, Invitrogen). Human and PCR primers were available in Supplementary table 8.

The copy numbers of the H1N1 NP viral RNA in lungs of virus-challenged WT and *db/db* mice were determined using the following primers, *Np* sense 5’-TGTCHTTCCAGGGGCGGGG-3’, *Np* antisense 5’- GTCAAARGARGGCACGATCGGG-3’ (Supplementary table 8). The relative expression level of target genes was calculated with normalization to β-actin values using the Ct method.

### Statistical analysis

Data were analysed with GraphPad Prism (version 7.0, GraphPad Software). Comparison of antibody response rates in low leptin and sufficient leptin groups were calculated by Chi-Square tests. WT and *db/db*, or *Cd4-Cre:LepR*^*+/+*^ and *Cd4-Cre:LepR*^*fl/fl*^ mice with immunisation and influenza virus infection, naïve CD4^+^ T cells with or without leptin treatment were analysed by non-parametric two-sided Mann–Whitney U-tests. The survival rate of H1N1 influenza virus-challenged mice were analysed with a Log-rank test. Different dose of leptin treatment, human T_FH_ cell culture, survival rate and antibody titters in mice with Fed, Fast + PBS, Fast + Leptin were analysed with one-way ANOVA or two-way ANOVA. *P*-value <0.05 was considered statistically significant.

### Reporting summary

Further information on research design is available in the Nature Research Reporting Summary linked to this article.

## Supplementary information

Supplementary Information

Reporting Summary

## Data Availability

All data are provided in the article and its Supplementary files or from the corresponding author upon reasonable request. Source data are provided with this paper.

## Source data

Source Data

## References

[1] Vinuesa CG, Linterman MA, Yu D, MacLennan IC (2016). Follicular helper T cells. Annu. Rev. Immunol..

[2] Crotty S (2014). T follicular helper cell differentiation, function, and roles in disease. Immunity.

[3] Ma CS (2012). Functional STAT3 deficiency compromises the generation of human T follicular helper cells. Blood.

[4] Ueno H, Banchereau J, Vinuesa CG (2015). Pathophysiology of T follicular helper cells in humans and mice. Nat. Immunol..

[5] Deng J (2018). Signal transducer and activator of transcription 3 hyperactivation associates with follicular helper T cell differentiation and disease activity in rheumatoid arthritis. Front. Immunol..

[6] Baumjohann D (2013). Persistent antigen and germinal center B cells sustain T follicular helper cell responses and phenotype. Immunity.

[7] He J (2013). Circulating precursor CCR7(lo)PD-1(hi) CXCR5(+) CD4(+) T cells indicate Tfh cell activity and promote antibody responses upon antigen reexposure. Immunity.

[8] Tangye SG, Ma CS, Brink R, Deenick EK (2013). The good, the bad and the ugly-TFH cells in human health and disease. Nat. Rev. Immunol..

[9] Goronzy JJ, Weyand CM (2013). Understanding immunosenescence to improve responses to vaccines. Nat. Immunol..

[10] Siegrist CA, Aspinall R (2009). B-cell responses to vaccination at the extremes of age. Nat. Rev. Immunol..

[11] Goodwin K, Viboud C, Simonsen L (2006). Antibody response to influenza vaccination in the elderly: a quantitative review. Vaccine.

[12] Bentebibel, S. E. et al. Induction of ICOS+CXCR3+CXCR5+ TH cells correlates with antibody responses to influenza vaccination. *Sci. Transl. Med.* **5**, 176ra32 (2013). 10.1126/scitranslmed.3005191 PMC3621097 23486778

[13] Herati RS (2017). Successive annual influenza vaccination induces a recurrent oligoclonotypic memory response in circulating T follicular helper cells. Sci. Immunol..

[14] Koutsakos M (2018). Circulating TFH cells, serological memory, and tissue compartmentalization shape human influenza-specific B cell immunity. Sci. Transl. Med..

[15] Considine RV (1996). Serum immunoreactive-leptin concentrations in normal-weight and obese humans. N. Engl. J. Med..

[16] Schultz BT (2016). Circulating HIV-specific interleukin-21(+)CD4(+) T cells represent peripheral Tfh cells with antigen-dependent helper functions. Immunity.

[17] Spensieri F (2013). Human circulating influenza-CD4+ ICOS1+IL-21+ T cells expand after vaccination, exert helper function, and predict antibody responses. Proc. Natl Acad. Sci. USA.

[18] Buck MD, Sowell RT, Kaech SM, Pearce EL (2017). Metabolic instruction of immunity. Cell.

[19] Ganeshan K, Chawla A (2014). Metabolic regulation of immune responses. Annu. Rev. Immunol..

[20] Osborn O, Olefsky JM (2012). The cellular and signaling networks linking the immune system and metabolism in disease. Nat. Med..

[21] Li S (2017). Metabolic phenotypes of response to vaccination in humans. Cell.

[22] Abella V (2017). Leptin in the interplay of inflammation, metabolism and immune system disorders. Nat. Rev. Rheumatol..

[23] Naylor C, Petri WA (2016). Leptin regulation of immune responses. Trends Mol. Med..

[24] Fonseca VR (2017). Human blood Tfr cells are indicators of ongoing humoral activity not fully licensed with suppressive function. Sci. Immunol..

[25] Ahima RS, Flier JS (2000). Leptin. Annu. Rev. Physiol..

[26] Lord GM (1998). Leptin modulates the T-cell immune response and reverses starvation-induced immunosuppression. Nature.

[27] Deng J (2012). Leptin exacerbates collagen-induced arthritis via enhancement of Th17 cell response. Arthritis Rheum..

[28] Yu Y (2013). Cutting edge: Leptin-induced RORgammat expression in CD4+ T cells promotes Th17 responses in systemic lupus erythematosus. J. Immunol..

[29] Schmitt N (2014). The cytokine TGF-beta co-opts signaling via STAT3-STAT4 to promote the differentiation of human TFH cells. Nat. Immunol..

[30] Asrir A, Aloulou M, Gador M, Perals C, Fazilleau N (2017). Interconnected subsets of memory follicular helper T cells have different effector functions. Nat. Commun..

[31] Ryg-Cornejo V (2016). Severe malaria infections impair germinal center responses by inhibiting T follicular helper cell differentiation. Cell Rep..

[32] Linterman MA (2010). IL-21 acts directly on B cells to regulate Bcl-6 expression and germinal center responses. J. Exp. Med..

[33] Nurieva RI (2008). Generation of T follicular helper cells is mediated by interleukin-21 but independent of T helper 1, 2, or 17 cell lineages. Immunity.

[34] Vogelzang A (2008). A fundamental role for interleukin-21 in the generation of T follicular helper cells. Immunity.

[35] Zotos D (2010). IL-21 regulates germinal center B cell differentiation and proliferation through a B cell-intrinsic mechanism. J. Exp. Med..

[36] Lam QL, Wang S, Ko OK, Kincade PW, Lu L (2010). Leptin signaling maintains B-cell homeostasis via induction of Bcl-2 and Cyclin D1. Proc. Natl Acad. Sci. USA.

[37] Reis BS (2015). Leptin receptor signaling in T cells is required for Th17 differentiation. J. Immunol..

[38] Deng J, Wei Y, Fonseca VR, Graca L, Yu D (2019). T follicular helper cells and T follicular regulatory cells in rheumatic diseases. Nat. Rev. Rheumatol..

[39] Wing JB, Tekguc M, Sakaguchi S (2018). Control of germinal center responses by T-follicular regulatory cells. Front. Immunol..

[40] La Cava A, Matarese G (2004). The weight of leptin in immunity. Nat. Rev. Immunol..

[41] Yang J (2016). Critical roles of mTOR Complex 1 and 2 for T follicular helper cell differentiation and germinal center responses. elife.

[42] Zeng H (2016). mTORC1 and mTORC2 kinase signaling and glucose metabolism drive follicular helper T cell differentiation. Immunity.

[43] Hao Y (2018). The kinase complex mTOR complex 2 promotes the follicular migration and functional maturation of differentiated follicular helper CD4(+) T cells during viral infection. Front. Immunol..

[44] Sallusto F, Lanzavecchia A, Araki K, Ahmed R (2010). From vaccines to memory and back. Immunity.

[45] Siegrist, C.-A. in *Vaccines* 5th edn. (eds. Plotkin, S., Orenstein, W. & Offit, P.) 17–36 (Saunders Elsevier, 2008).

[46] De Rosa V (2007). A key role of leptin in the control of regulatory T cell proliferation. Immunity.

[47] Procaccini C (2016). The proteomic landscape of human ex vivo regulatory and conventional T cells reveals specific metabolic requirements. Immunity.

[48] Procaccini C (2010). An oscillatory switch in mTOR kinase activity sets regulatory T cell responsiveness. Immunity.

[49] Rivadeneira DB (2019). Oncolytic viruses engineered to enforce leptin expression reprogram tumor-infiltrating T cell metabolism and promote tumor clearance. Immunity.

[50] Wang Z (2019). Paradoxical effects of obesity on T cell function during tumor progression and PD-1 checkpoint blockade. Nat. Med.

[51] Zhang C (2019). STAT3 activation-induced fatty acid oxidation in CD8(+) T effector cells is critical for obesity-promoted breast tumor growth. Cell Metab..

[52] Wehrens A, Aebischer T, Meyer TF, Walduck AK (2008). Leptin receptor signaling is required for vaccine-induced protection against Helicobacter pylori. Helicobacter.

[53] Lourenco EV, Liu A, Matarese G, La Cava A (2016). Leptin promotes systemic lupus erythematosus by increasing autoantibody production and inhibiting immune regulation. Proc. Natl Acad. Sci. USA.

[54] Wang M (2018). Leptin upregulates peripheral CD4(+)CXCR5(+)ICOS(+) T cells via increased IL-6 in rheumatoid arthritis patients. J. Interferon Cytokine Res..

[55] Ovsyannikova IG (2014). Leptin and leptin-related gene polymorphisms, obesity, and influenza A/H1N1 vaccine-induced immune responses in older individuals. Vaccine.

[56] Fischinger S, Boudreau CM, Butler AL, Streeck H, Alter G (2019). Sex differences in vaccine-induced humoral immunity. Semin. Immunopathol..

[57] Brodin P, Davis MM (2017). Human immune system variation. Nat. Rev. Immunol..

[58] Pulendran B (2014). Systems vaccinology: probing humanity’s diverse immune systems with vaccines. Proc. Natl Acad. Sci. USA.

[59] Linterman MA, Hill DL (2016). Can follicular helper T cells be targeted to improve vaccine efficacy?. F1000Res..

[60] Streeck H, D’Souza MP, Littman DR, Crotty S (2013). Harnessing CD4(+) T cell responses in HIV vaccine development. Nat. Med..

[61] Pilkinton MA (2017). Greater activation of peripheral T follicular helper cells following high dose influenza vaccine in older adults forecasts seroconversion. Vaccine.

[62] DiazGranados CA (2014). Efficacy of high-dose versus standard-dose influenza vaccine in older adults. N. Engl. J. Med..

[63] Gabriely I, Ma XH, Yang XM, Rossetti L, Barzilai N (2002). Leptin resistance during aging is independent of fat mass. Diabetes.

[64] Wang X (2016). IL-17A promotes pulmonary B-1a cell differentiation via induction of Blimp-1 expression during influenza virus infection. PLoS Pathog..

[65] Wei L, Laurence A, Elias KM, O’Shea JJ (2007). IL-21 is produced by Th17 cells and drives IL-17 production in a STAT3-dependent manner. J. Biol. Chem..

