# Supplementary material for: The metabolic hormone leptin promotes the function of TFH cells and supports vaccine responses
Source: Nat Commun. 2021 May 24;12:3073. doi: 10.1038/s41467-021-23220-x (PMC8144586; doi:10.1038/s41467-021-23220-x)
Supplement: Supplementary file 1 — Supplementary Information [file 41467_2021_23220_MOESM1_ESM.pdf]

Deng *et al*.

The metabolic hormone leptin promotes the function of T_{FH} cells and associates with vaccine responses

Supplementary Figures 1-15

Supplementary Tables 1-8

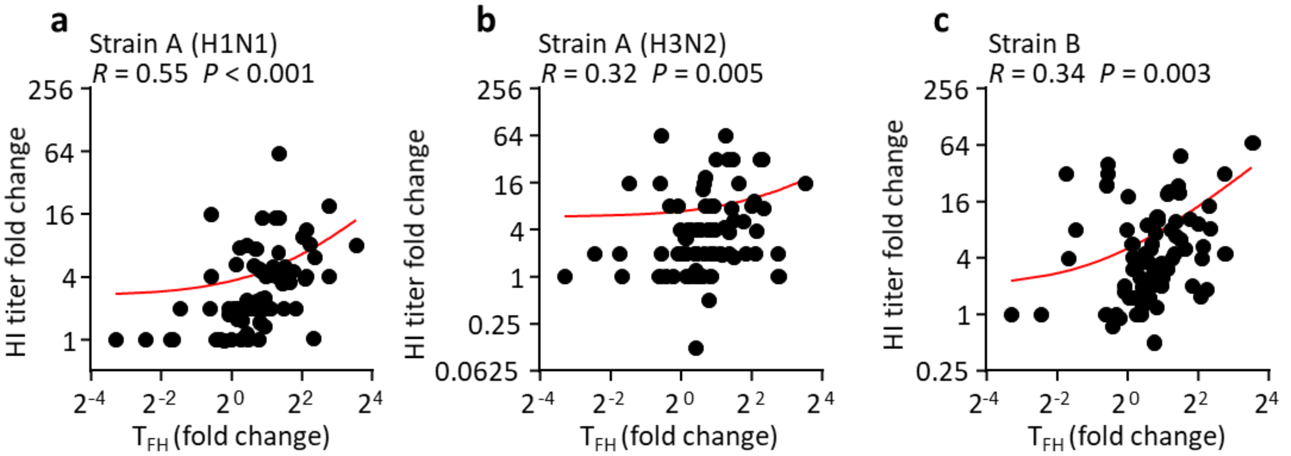

Supplementary Figure 1. T_{FH} activities correlate with antibody titters after flu vaccination in adults

a-c, The correlation of circulating T_{FH} (cT_{FH}) fold change (day7/day0) and antibody titter fold change in strain A (**a**) (H1N1), (**b**) (H3N2) and (**c**) stain B in healthy adults (aged 18-60 years, $n = 76$) immunized with the 2015/2016, 2016/2017 seasonal influenza vaccine. The cT_{FH} cells were gated as $CD4^{+}CD45RA^{-}CXCR5^{+}PD\text{-}1$ or $CD4^{+}CXCR5^{+}PD1^{+}ICOS^{+}$ as described in previous publications [14, 25], and the fold changes on day 7 were calculated by dividing values at day 0.

Data were analysed by Pearson's correlation coefficients (**a-c**). Cohorts are from **Figure. 1a**.

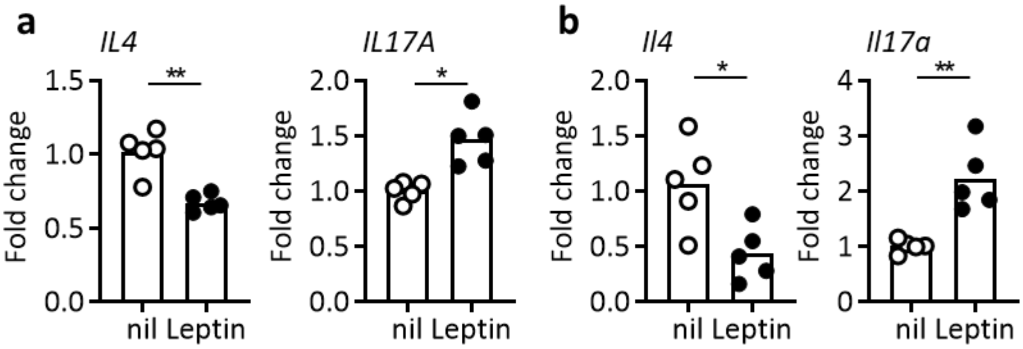

Supplementary Figure 2. Leptin inhibits *IL4* and increases *IL17* transcripts in activated human and mouse naïve CD4^{+} T cells

a,**b**, Real-time PCR analysis of *IL4* and *IL17* in naïve CD4^{+} T cells from PBMC of healthy controls with anti-CD3/CD28 activation for 3 days, and further treated with leptin for 12 hours (**a**) (*IL4*: $^{**}P$ = 0.0027, *IL17A*: $^{*}P$ = 0.0156), and mouse naïve CD4^{+} T cells with anti-CD3/CD28 activation for 2 days, and further treated with leptin for 12 hours (**b**) (*Il4*: $^{**}P$ = 0.0260, *Il17a*: $^{**}P$ = 0.0045). Data are shown for individual (dots, n = 5) and mean (bars) values, and analysed by Mann-Whitney U-test (**a**, **b**). *: $P < 0.05$, **: $P < 0.01$. Results are representative of three independent experiments.

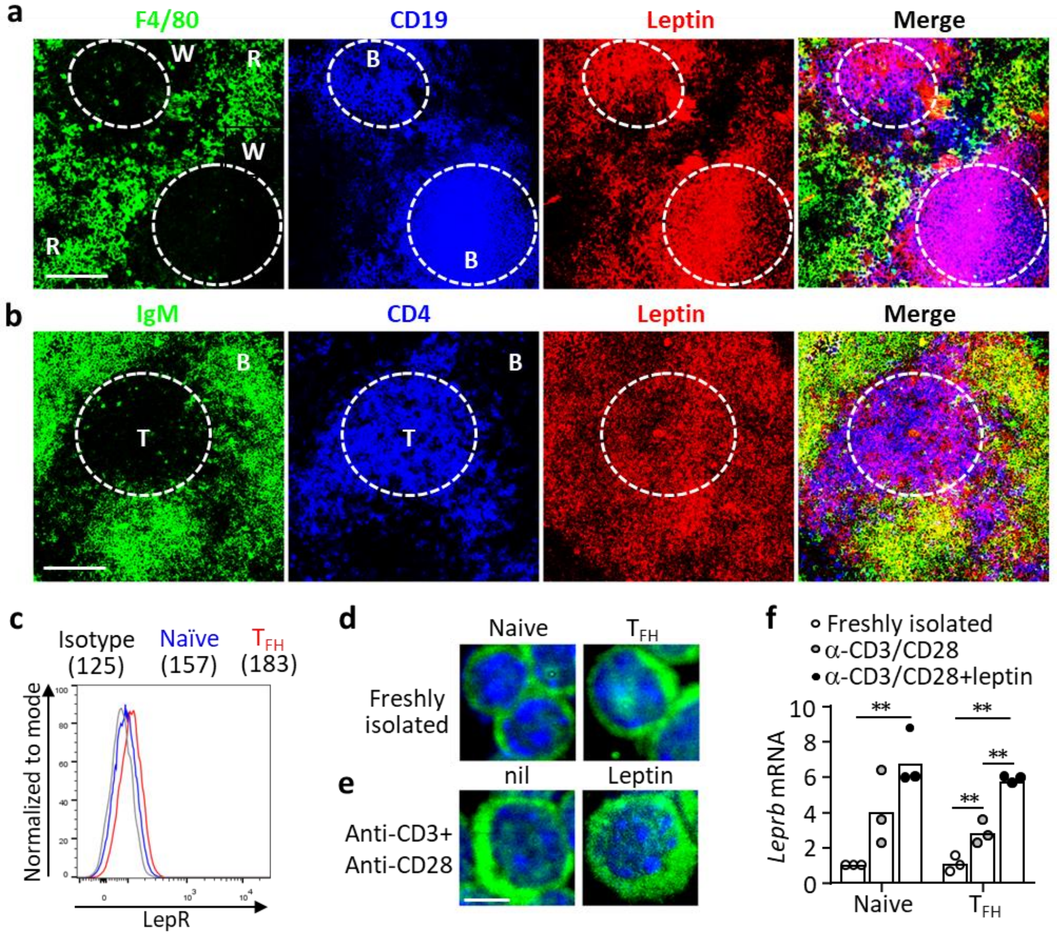

Supplementary Figure 3. Leptin expression in B follicles and LepR expression in T_{FH} cells

a,**b**, Representative immunofluorescence (IF) staining of leptin (red) in B cell follicles (CD19, blue) but not in the red pulp (F4/80, green) (**a**), leptin (red) in B cell follicles (IgM, green) and T cell zone (CD4, Blue), B: B cell follicles (IgM^+), T: T cell zone ($CD4^+$) (**b**) of spleens from WT mice infected with H1N1 for 9 days. Abbreviations: W: white pulp ($F4/80^-$); R: red pulp ($F4/80^+$); B: B cell follicles ($CD19^+$); T: T cell zone ($CD4^+$). Scale bar = 50μm.

c, Representative FACS histograms showing LepR expression on $CD4^+CD44^-CD62L^+$ naive T and $CD4^+CD44^+CD62L^-CXCR5^+PD\text{-}1^+$ T_{FH} cells from WT mice 9 days post H1N1 influenza virus challenge.

d,**e**, Representative IF staining of LepR expression (green) on naive $CD4^+$ T, T_{FH} cells (**d**), and naïve $CD4^+$ T cells stimulated with anti-CD3/CD28 and leptin for 12 hours (**e**). Nuclei were labelled using DAPI (blue). Scale bar = 10μm.

f, Real-time PCR analysis of *Leprb* in naive $CD4^+$ T cells and T_{FH} cells stimulated with anti-CD3/CD28 and leptin (200 ng/mL) for 3 hours (Naive: $^{**}P = 0.0073$; T_{FH}: α-CD3/CD28 vs Freshly isolated: $^{**}P = 0.0016$, α-CD3/CD28+leptin vs Freshly isolated:$^{**}P < 0.0001$, leptin: $^{**}P < 0.0058$).

Data are shown for individual (dots, $n = 3$) and mean (bars) values analysed by two-way ANOVA (**f**). **: $P < 0.01$. Results are representative of three independent experiments.

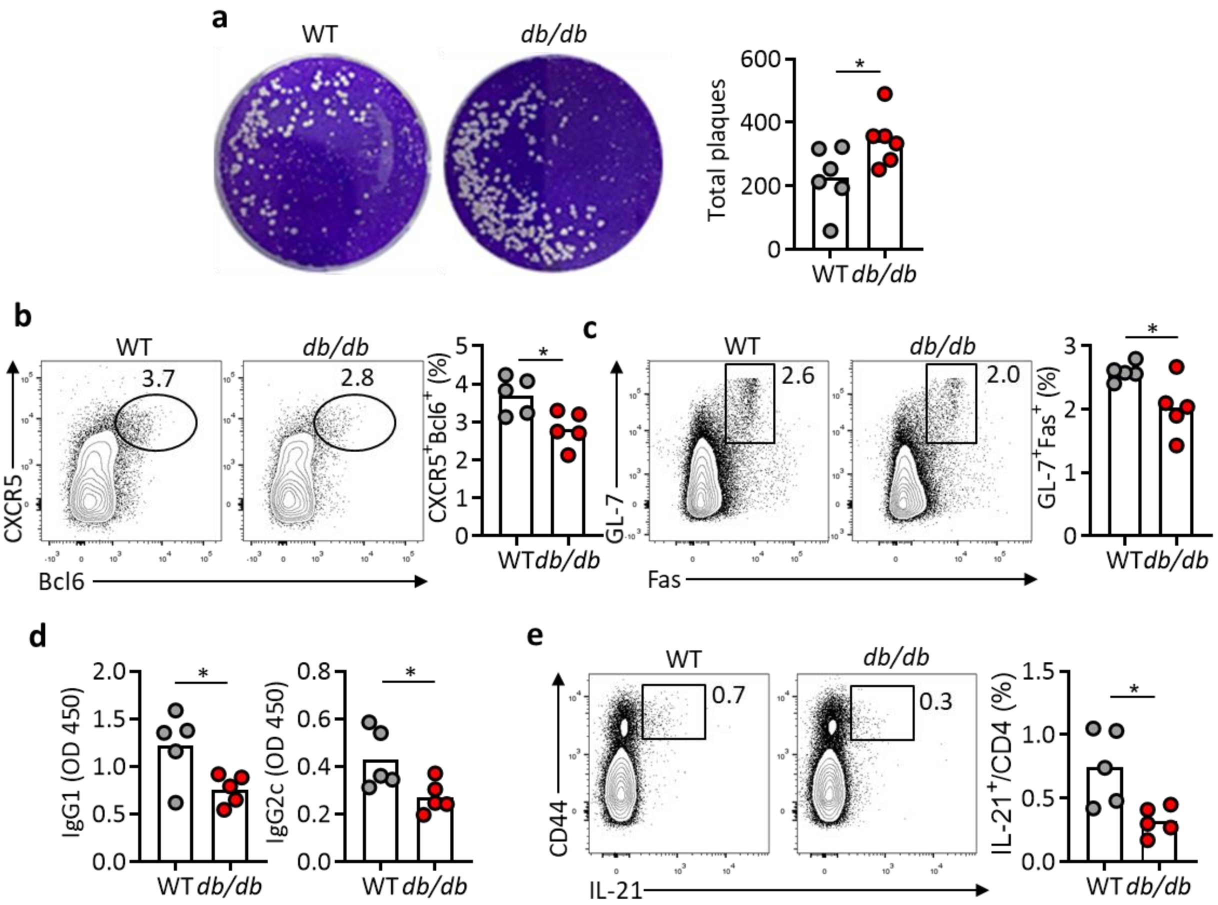

Extended Figure 4. LepR deficiency impairs T_{FH} and B cells responses in infection and TNP-KLH immunization

a, H1N1 viral titter in the lung tissues of WT and *db/db* mice (dots, *n* = 6 per genotype) 9 days post H1N1 influenza viral infection were determined by plaque assay (**P* = 0.0390).

b,c, WT and *db/db* mice (dots, *n* = 5 per genotype) were immunised (s.c.) with TNP-KLH/CFA. Representative FACS plots showing $CXCR5^+Bcl6^+$ T_{FH} (**b**) (**P* = 0.0493), $Fas^+GL\text{-}7^+$ GC B cells (**c**) (**P* = 0.0251) in the draining lymph nodes 9 days post immunisation.

d, ELISA measurement of anti-TNP IgG1 (**P* = 0.0481) and IgG2c (**P* = 0.0153) titters in sera (dots, *n* = 5 per genotype).

e, Representative FACS plots showing IL-21 ratio in $CD4^+CD44^+$ T cells in the draining lymph nodes 9 days post immunization (**P* = 0.0300) (dots, *n* = 5 per genotype).

Data are shown for individual (dots) and mean (bars) values and analyzed by Mann-Whitney U-test (**a**-**e**). *: $P < 0.05$. Results are representative of three independent experiments.

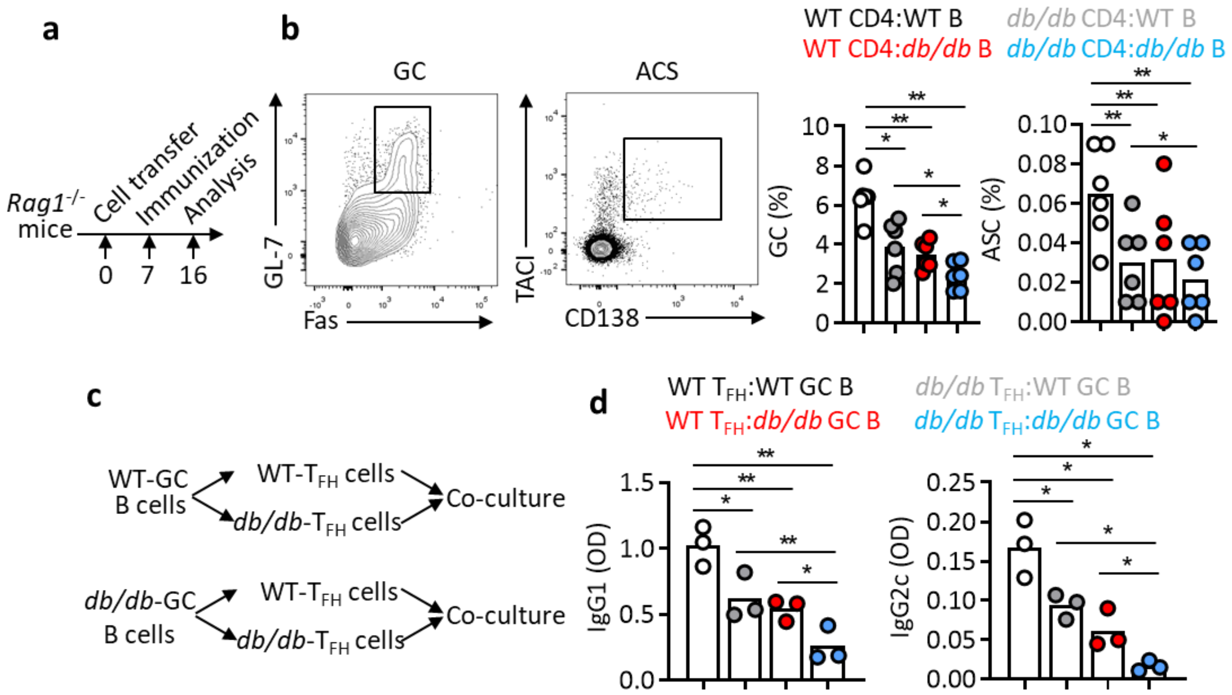

Supplementary Figure 5. LepR signals in both T and B cells are required for optimal antibody responses

a, Schematic illustration of cell transfer, immunization and analysis. Naïve $CD4^+$ T cells (3×10^6) and B cells (3×10^6) isolated from WT or *db/db* mice were mixed into 4 groups (WT T cells + WT B cells; *db/db* T cells + WT B cells; WT T cells + *db/db* B cells; *db/db* T cells + *db/db* B cells) and adoptively transferred into *Rag1*$^{-/-}$ recipients (dots, *n* = 6 per group). After 7 days of transfer, mice were immunized (s.c.) with NP-OVA emulsified in CFA. Germinal centre (GC) B cells and antibody-secreting cells (ASCs) in spleens of *Rag1*$^{-/-}$ recipients were analysed 9 days post cell transfer.

b, Flow cytometric analysis and statistics of $B220^+Fas^+GL\text{-}7^+$ GC B cells (WT CD4:WT B vs *db/db* CD4:WT B, **P* = 0.0162; WT CD4:WT B vs WT CD4: *db/db* B, **P* = 0.0023; WT CD4:WT B vs *db/db* CD4: *db/db* B, **P* = 0.0003; *db/db* CD4:WT B vs *db/db* CD4: *db/db* B, **P* = 0.0218; WT CD4: *db/db* B vs *db/db* CD4: *db/db* B, **P* = 0.0089;) (dots, *n*=6 per group), and $B220^-CD138^+TACI^+$ ASCs (WT CD4:WT B vs *db/db* CD4:WT B, **P* = 0.0005; WT CD4:WT B vs WT CD4: *db/db* B, **P* = 0.0012; WT CD4:WT B vs *db/db* CD4: *db/db* B, **P* = 0.0004; *db/db* CD4:WT B vs *db/db* CD4: *db/db* B, **P* = 0.0456) (dots, *n*=6 per group). in recipient *Rag1*$^{-/-}$ mice 9 days post NP-OVA/CFA immunisation

c, Experimental design scheme of T_{FH} and B cell coculture. $B220^-CD4^+CD25^-CD44^+CD62L^-CXCR5^+PD\text{-}1^+$ T_{FH} cells and $B220^+Fas^+GL\text{-}7^+$ GC B cells from WT and *db/db* mice with NP-OVA/CFA immunisation (s.c.) were sorted and cocultured with the addition of NP-OVA (5 μg/ml) for 9 days.

d, ELISA measurement of anti-NP specific IgG1 (WT T_{FH}:WT GC B vs *db/db* T_{FH}:WT GC B, **P* = 0.0369; WT T_{FH}:WT GC B vs WT T_{FH}:*db/db* GC B, **P* = 0.0041; WT T_{FH}:WT GC B vs *db/db* T_{FH}:*db/db* GC B, **P* = 0.0094; *db/db* T_{FH}:WT GC B vs *db/db* T_{FH}:*db/db* GC B, **P* = 0.0022; WT T_{FH}:*db/db* GC B vs *db/db* T_{FH}:*db/db* GC B, **P* = 0.0280) and IgG2c titters (WT T_{FH}:WT GC B vs *db/db* T_{FH}:WT GC B, **P* = 0.0203; WT T_{FH}:WT GC B vs WT T_{FH}:*db/db* GC B, **P* = 0.0453; WT T_{FH}:WT GC B vs *db/db* T_{FH}:*db/db* GC B, **P* = 0.0124; *db/db* T_{FH}:WT GC B vs *db/db* T_{FH}:*db/db* GC B, **P* = 0.0138; WT T_{FH}:*db/db* GC B vs *db/db* T_{FH}:*db/db* GC B, **P* = 0.0252) in the supernatant of cultured T_{FH}:B cells (dots, *n* = 3 per group).

Data are shown for individual (dots) and mean (bars) values, and analysed by two-way ANOVA (**b,d**). *: P < 0.05, **: P < 0.01. Results are representative of three independent experiments.

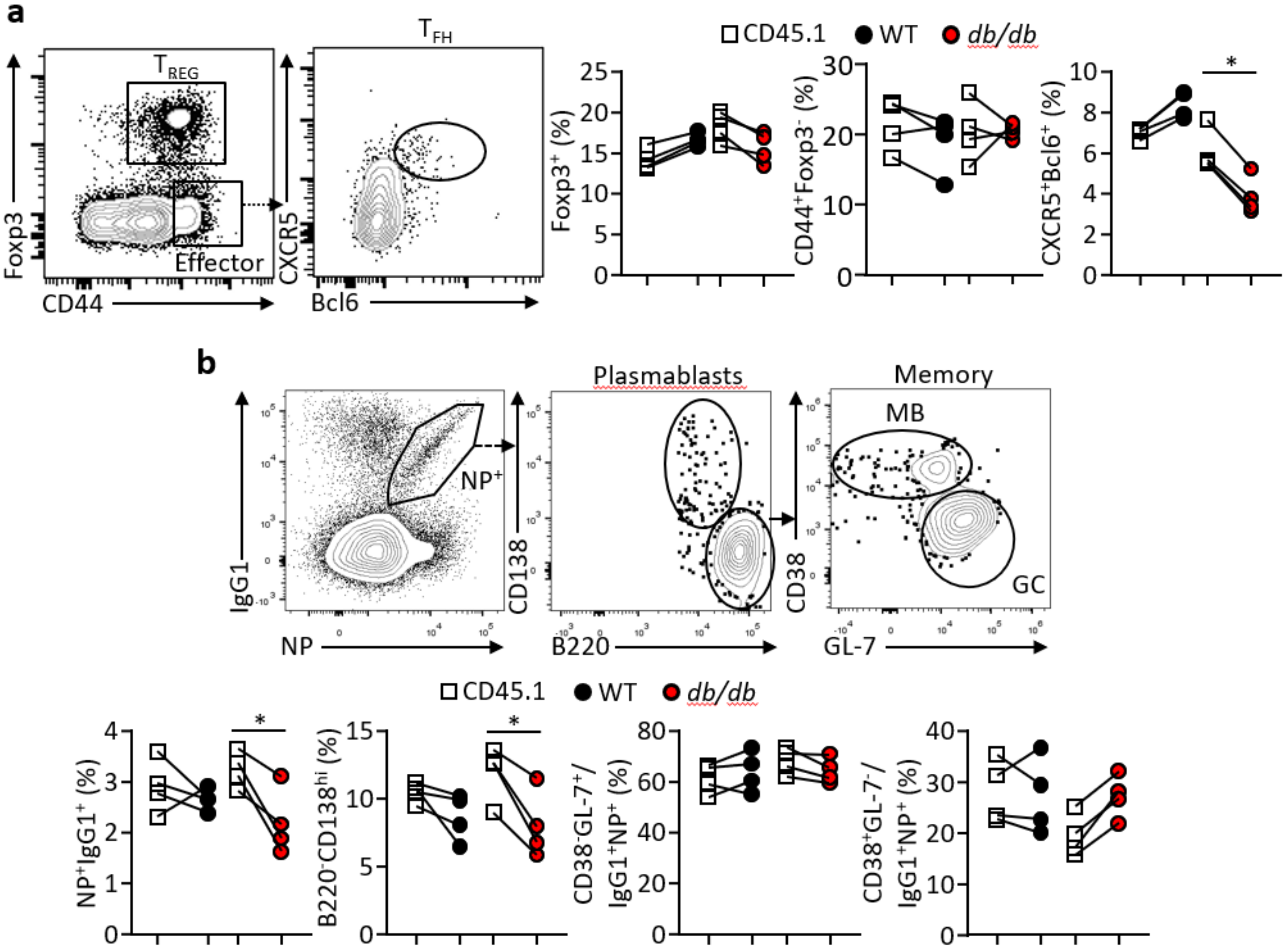

Supplementary Figure 6. Leptin intrinsically regulates B cells and T_{FH} responses

a,**b**, Representative FACS plots and statistics showing Foxp3$^+$ T_{REG} cells, CD44^{high}Foxp3$^-$ effector cells and CXCR5$^+$Bcl6$^+$ T_{FH} cells (**a**) (CD45.1 vs *db/db*: *P = 0.0110), NP$^+$IgG1$^+$ (CD45.1 vs *db/db*: *P = 0.0274), CD138^{high} ASCs (CD45.1 vs *db/db*: *P = 0.0231), CD38$^+$GL-7$^-$ memory B, CD38$^-$GL-7$^+$ GC B cells (**b**) in spleens from mixed WT and *db/db* BM chimaeras with s.c. NP-OVA/CFA immunisation (dots, n = 4 per group).

Data are shown for individual (dots), and analyzed by one-way ANOVA. *: P < 0.05. Results are representative of two independent experiments.

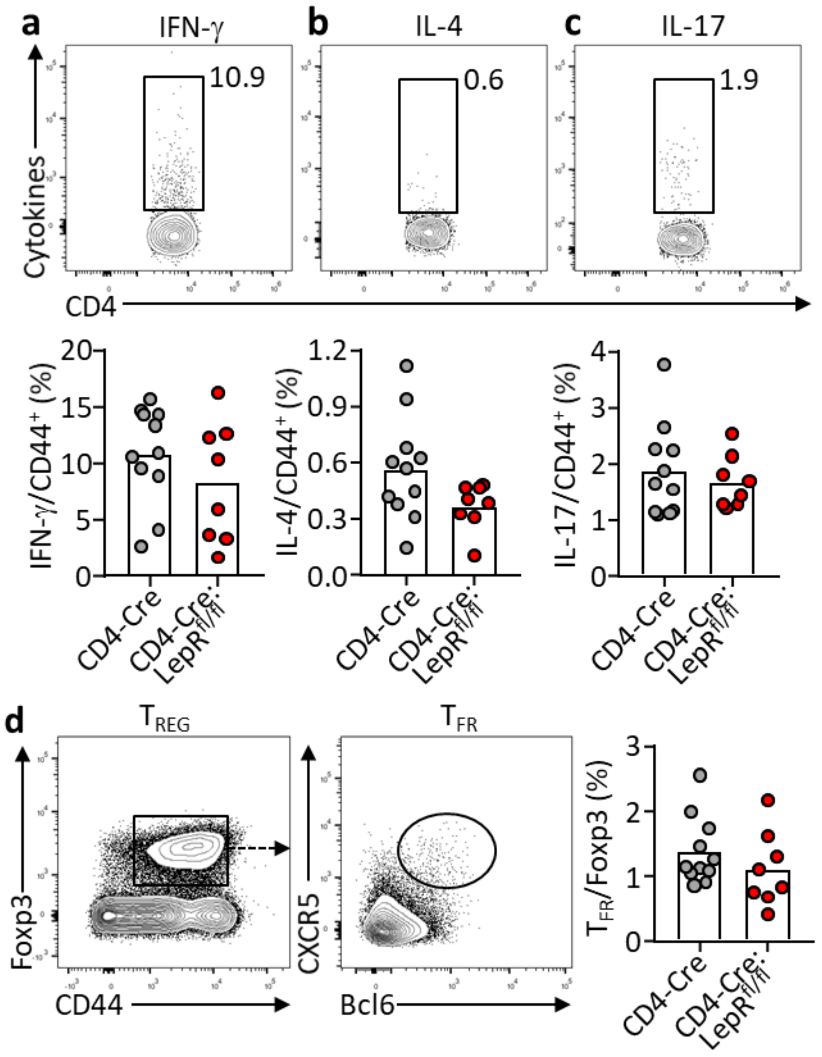

Supplementary Figure 7. LepR signalling shows negligible effect on the production of IFN-γ, IL-4 and IL-17 in CD4-Cre:*LepR*$^{fl/fl}$ mice with influenza virus infection

a-**d**, CD4-Cre:*LepR*$^{+/+}$ mice (*n*=8) and CD4-Cre:*LepR*$^{fl/fl}$ mice (*n* = 11) were infected with influenza virus. Mediastinal lymph nodes were analysed at day 9 post infection. Representative FACS plots and statistics showing IFN-γ (**a**), IL-4 (**b**) and IL-17 (**c**) in CD4^{+} T cells, and CXCR5^{+}Foxp3^{+}Bcl6^{+} T_{FR} cells (**d**).

Data are shown for individual (dots) and mean (bars) values and analysed by Mann-Whitney U-test. Results are representative of two independent experiments.

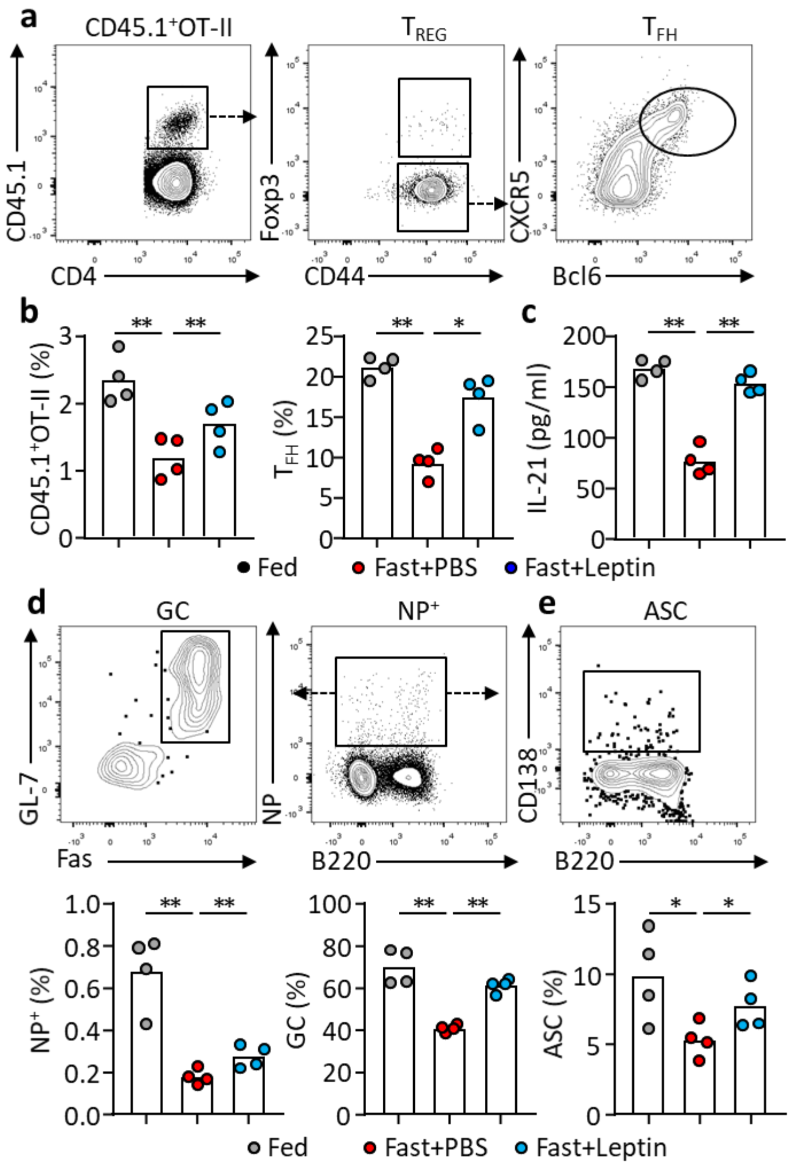

Supplementary Figure 8. Leptin supplementation compensates fasting-reduced antigen-specific T_{FH} cells and antigen-specific B cell responses

a-e, $B220^-CD4^+CD25^-CD44^-CD62L^+$ naïve T cells from $CD45.1^+$ OT-II mice were sorted and transferred (i.v.) to CD45.2 recipients on day -1, followed by NP-OVA/CFA immunisation on day 0. Mice were normal diet (Fed), acutely fasted with PBS (Fast+PBS) or leptin (Fast+Leptin) supplementation from day 7 to day 8, T_{FH} and B cells in the draining lymph nodes (dLNs) were analysed on day 9. Flow cytometric analysis and statistics of $CD45.1^+$ OT-II T cells and $CXCR5^+Bcl6^+$ T_{FH} cells (**a, b**) (CD45.1+OT-II: $^{**}P = 0.0044$, $^{**}P = 0.0066$; T_{FH}: $^{**}P = 0.0011$, $^{*}P = 0.0110$; IL-21: $^{**}P = 0.0005$, $^{**}P = 0.0003$), NP^+ GC B and ASCs (**d, e**) (NP: $^{**}P = 0.0088$, $^{**}P = 0.0071$; GC: $^{**}P = 0.0048$, $^{**}P = 0.0006$; GC: $^{*}P = 0.0209$, $^{*}P = 0.0337$) in the dLNs, and ELISA measurement of serum IL-21 levels (**c**) 9 days post immunisation ($n = 4$).

Data are shown for individual (dots) and mean (bars) values and analysed by two-way ANOVA. *: $P < 0.05$, **: $P < 0.01$. Results are representative of two independent experiments.

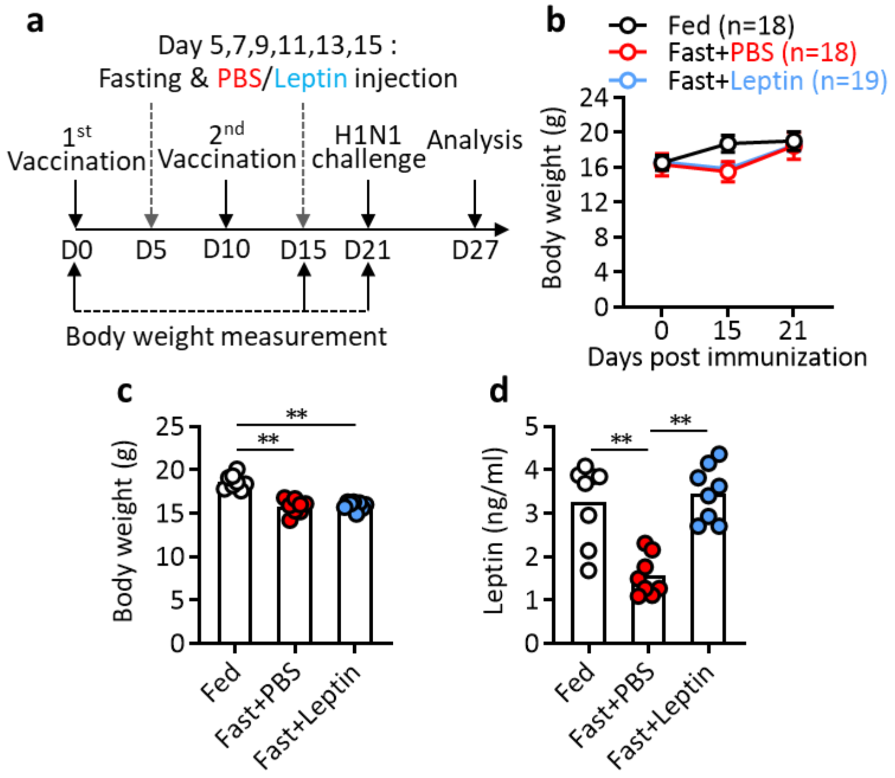

Supplementary Figure 9. Acute fasting temporarily reduces body weights and leptin levels

a, Experiment design and mice body weight measurement on day 0, 15 and 21. Same experimental scheme are shown in **Figure. 7a**.

b, Kinetics of body weight changes on day 0, 15 and 21 (bars: mean±SEM).

c,d, Bodyweight (**c**) (Fed vs Fast+PBS: ***P* = 0.0010, Fed vs Fast+leptin: ***P* < 0.0001) and serum leptin levels (**d**) (***P* = 0.0005, ***P* = 0.0003) on day 15 (dots, *n* = 8 per group).

Data are shown as mean and standard error of the mean±SEM, and analysed by two-way ANOVA. **: *P* < 0.01. Results are representative of two independent experiments.

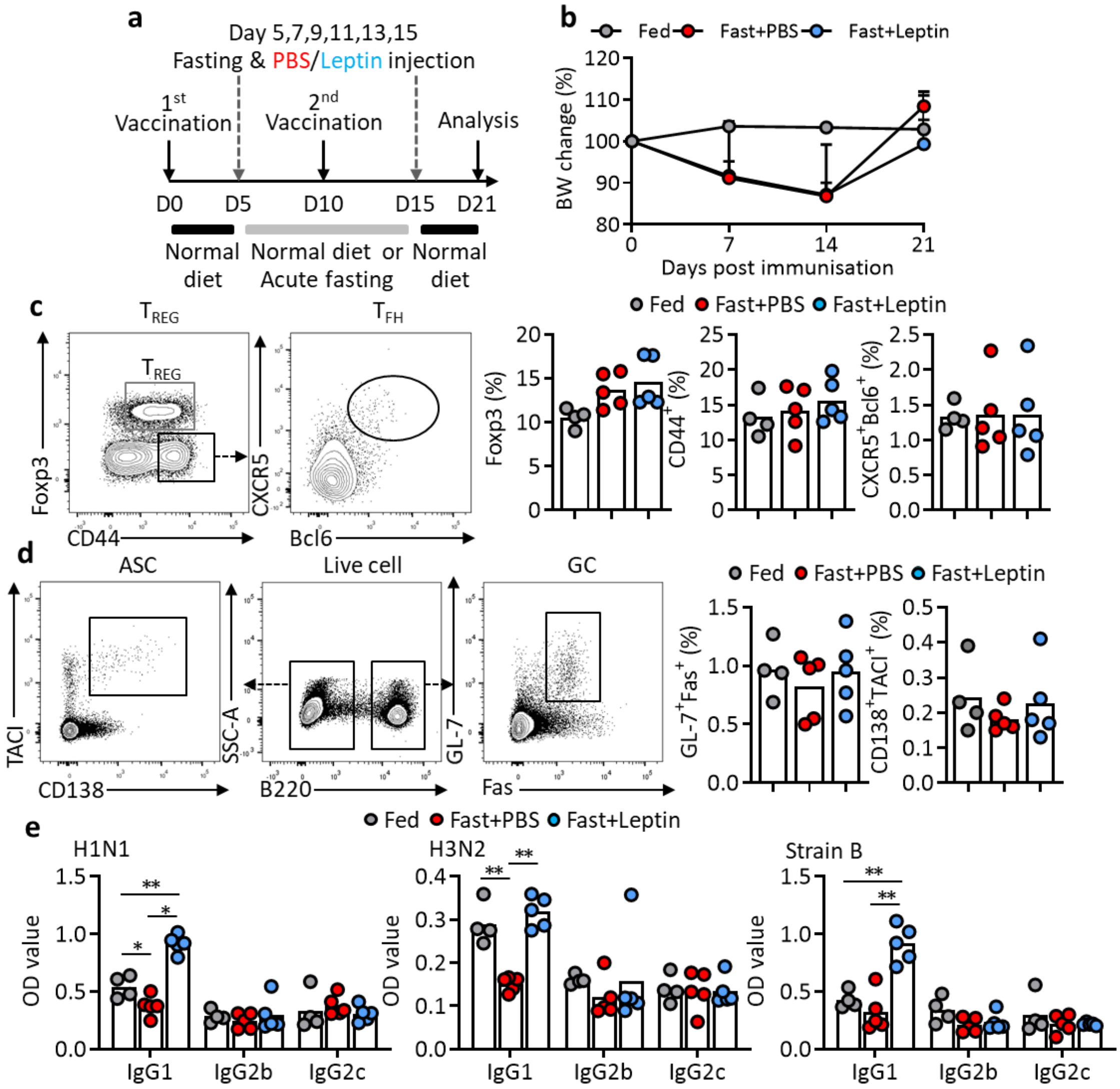

Supplementary Figure 10. Starving and leptin supplementation show negligible effects of GC and T_{FH} response in CD4-Cre:LepR$^{fl/fl}$ mice with human flu vaccine immunisation

a, Female CD4-Cre:LepR$^{fl/fl}$ mice were immunized (s.c.) with human influenza vaccine (GSK, Fluarix^{TM}, Tetra, 2017-2018), following various feeding regimens: normal diet (Fed) (*n* = 4), fasting with PBS (Fast+PBS) (*n* = 5), fasting with leptin treatment (Fast+leptin) (*n* = 5) from day 5 to day 15, and switched to normal diet till day 21.

b, Kinetics of body weight (BW) changes (bars: mean±SEM).

c, d, Representative FACS plots and statistics showing Treg, CD44$^+$ and T_{FH} cells (**c**), GC B cells, ASCs (**d**) in spleens of mice with different feeding regimens.

e, ELISA measurement of H1N1-, H3N2- and strain B-specific IgG1 (H1N1: Fed vs Fast+PBS, **P* = 0.0135; Fed vs Fast+Leptin: **P* = 0.0004; PBS vs Leptin: **P* = 0.0003; H3N2: Fed vs Fast+PBS, **P* = 0.0042; PBS vs Leptin: **P* = 0.0008; Strain B: Fed vs Fast+Leptin: ***P* = 0.0018; PBS vs Leptin: ***P* = 0.0056;), IgG2b, and IgG2c titters in the serum of mice with different feeding regimens.

Data are shown for individual (dots) and mean (bars) values and analysed by two-way ANOVA (b). *: $P < 0.05$, **: $P < 0.01$. Results are representative of two independent experiments.

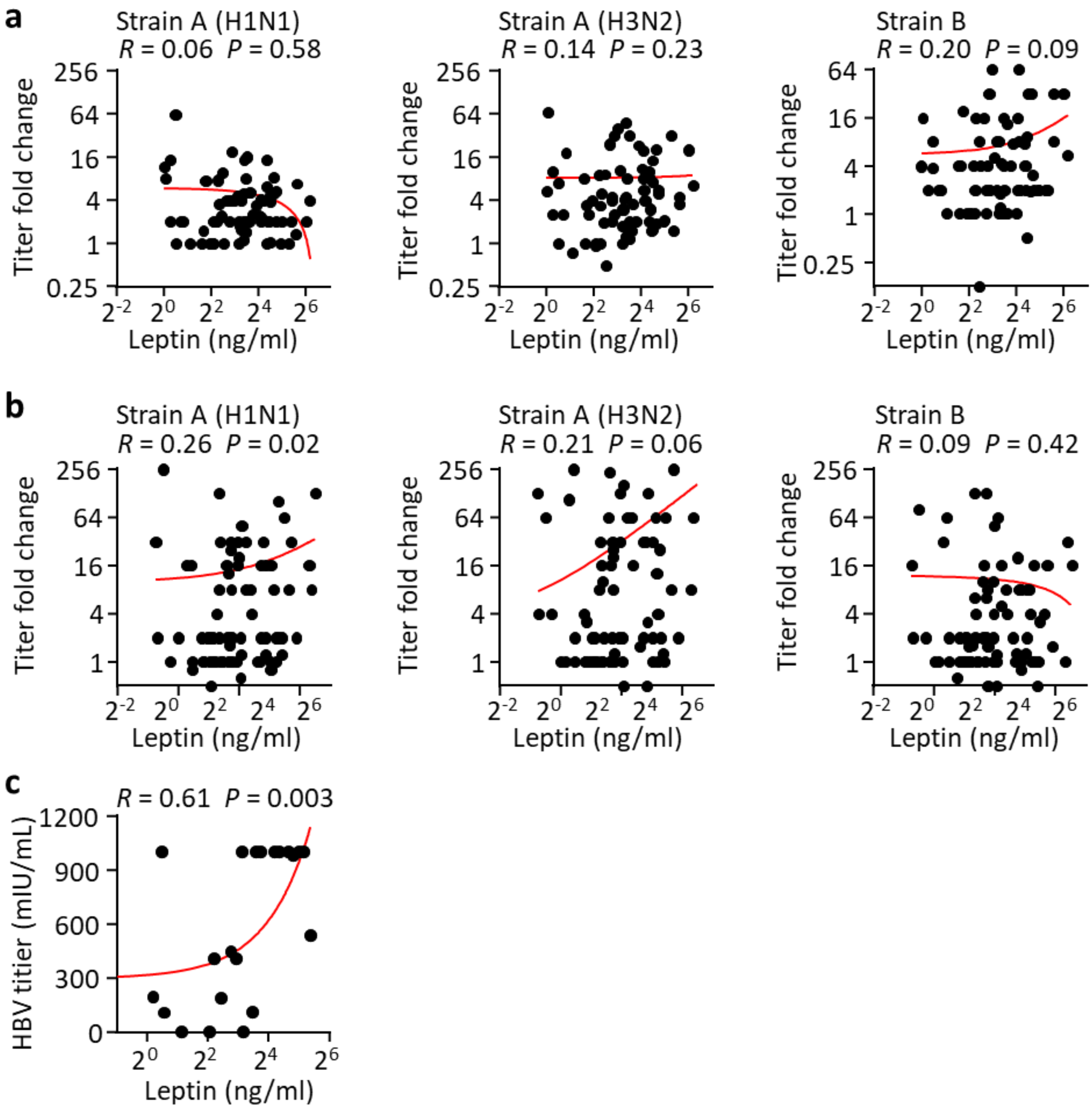

Supplementary Figure 11. Correlation between serum leptin levels and antibody titters after vaccinations

a, Correlation of serum leptin levels and HI titter fold change in strain A (H1N1), strain A (H3N2) and strain B in healthy young adults (*n* = 76) immunized with the seasonal influenza vaccine. The same cohort in **Fig. 1a**.

b, Correlation of serum leptin levels and HI titter fold change in strain A (H1N1), strain A (H3N2) and strain B in healthy elderly adults (*n* = 83) immunized with the seasonal influenza vaccine. The same cohort in **Fig. 1c**.

c, Correlation of serum leptin levels and antibody (Ab) titter post vaccine in young adults (*n* = 22) immunized with HBV vaccine. The same cohort in **Fig.1e**.

Data were analysed by Pearson's correlation coefficients. Cohorts are from **Fig. 1**.

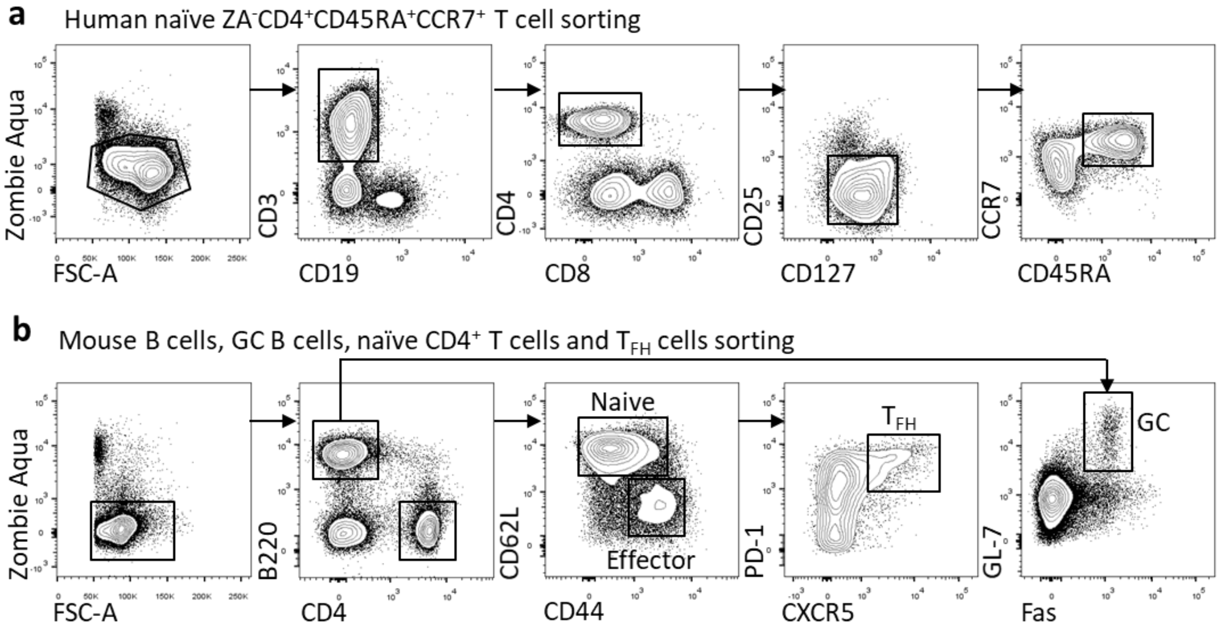

Supplementary Figure 12. Gating strategies used for cell sorting of naïve CD4+ T cells, T_{FH} and B cells

a, Gating strategy to sort human naïve $CD3^+CD4^+CD25^-CCR7^+CD45RA^+$ cells from PBMC of healthy donors presented on Fig. 2c-g.

b, Mouse $ZA^-B220^+CD4^-$ cells, $ZA^-B220^-CD4^+$ cells sorting presented on Fig S5a and Fig S8a, $CD4^+CD62L^+CD44^+$ naïve cells sorting in Fig 5 and Fig 6, $ZA^-B220^-CD4^+CD44^+CD62L^-CXCR5^+PD\text{-}1^+$ T_{FH} cells and $ZA^-B220^+GL\text{-}7^+Fas^+$ GC B cells on Fig 5a and Fig S5c.

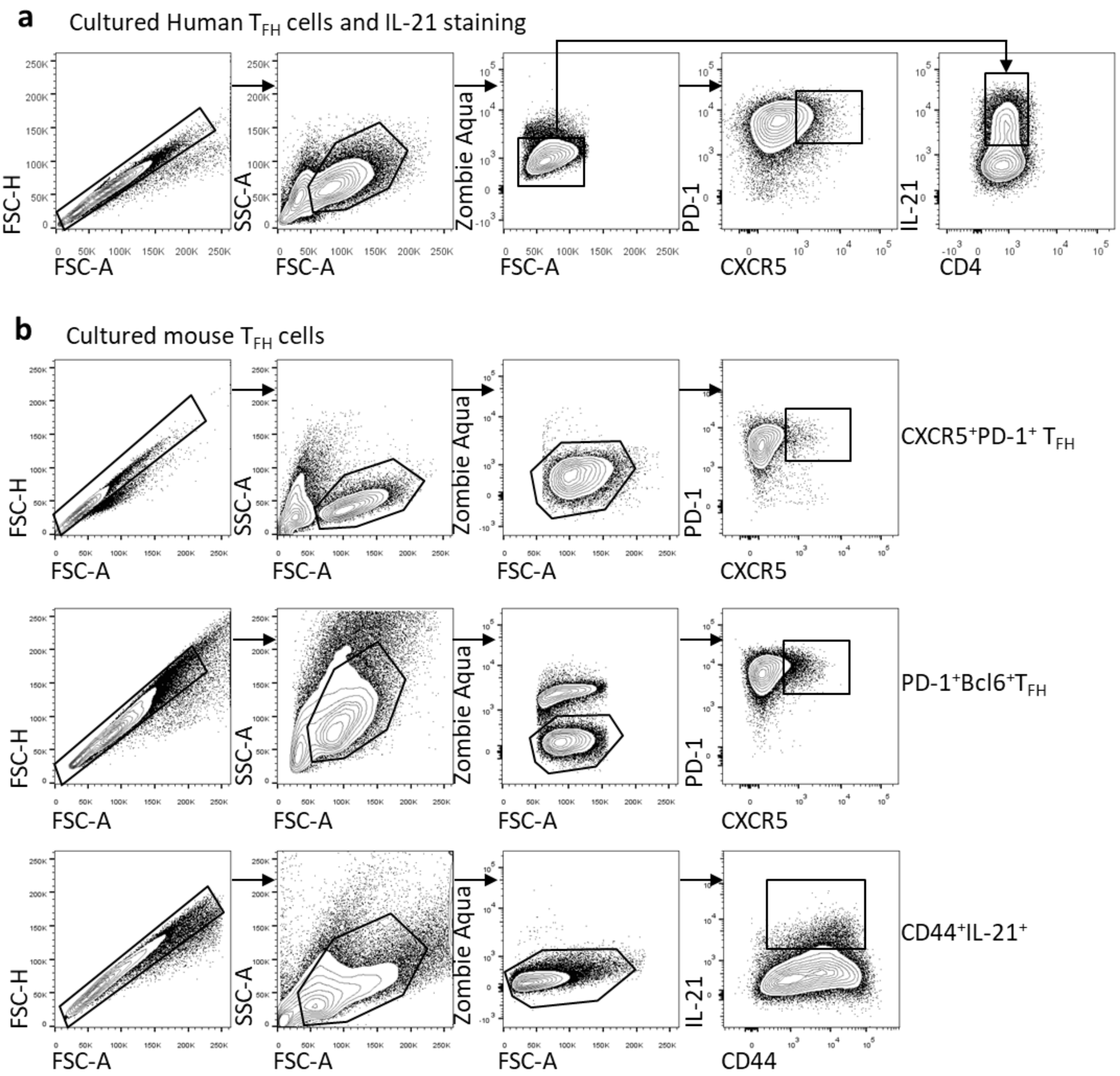

Supplementary Figure 13. **Gating strategies used for flow cytometric analysis of culture human and mouse T_{FH} cells**

a, Gating strategy to analyze cultured human CXCR5^{+}PD-1^{+} T_{FH} cells and IL-21 production with leptin treatment presented on Fig 2d-g.

b, Gating strategy to analyze cultured mouse CXCR5^{+}PD-1^{+} T_{FH} cells with leptin treatment presented on Fig 6b, PD-1^{+}Bcl6^{+} T_{FH} cells presented on Fig 6f-h, and CD44^{+}IL-21^{+} cells presented on Fig 6g.

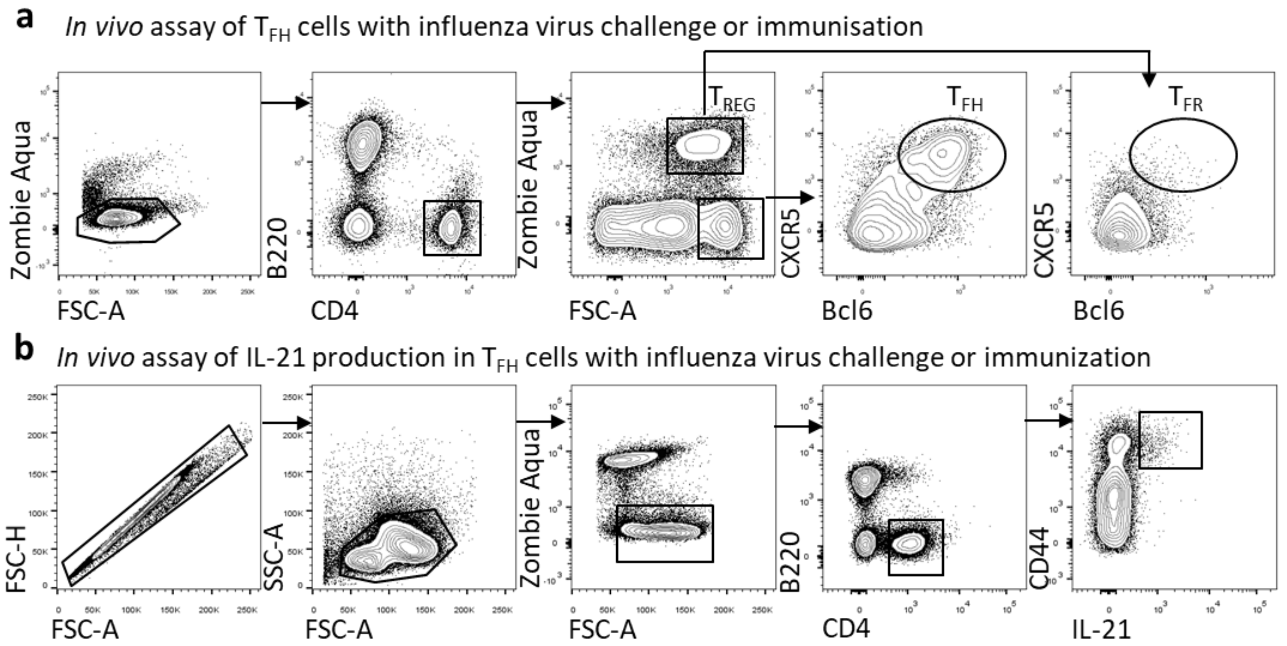

Supplementary Figure 14. **Gating strategies used for flow cytometric analysis of mouse T_{FH} cells in vivo**

a, Gating strategy to analyze $ZA^-B220^-CD4^+CD44^+Foxp3^-CXCR5^+Bcl6^+$ T_{FH} cells from WT and *db/db* mice 9 days post influenza viral challenge presented on Fig. 3d, *CD4-Cre:LepR*$^{+/+}$ and *CD4-Cre:LepR*$^{fl/fl}$ mice 9 days post influenza viral challenge presented on Fig. 4a, b, Fig Sd, and WT mice with vaccination-challenge model presented on Fig. 7f, WT and *db/db* mice 9 days post NP-OVA immunization presented on Fig S4b, *Rag1*$^{-/-}$ bone marrow chimeras from WT or *db/db* mice 9 days post NP-OVA immunization presented on Fig S6a, CD45.2 recipient mice transferred with CD45.1$^+$ OT-II naïve CD4+ T cells followed by fast and leptin treatment presented on Fig S8a, and *CD4-Cre:LepR*$^{fl/fl}$ mice immunized with human influenza vaccine on day 20 presented on Fig S10c.

b, Gating strategy to analyze $ZA^-B220^-CD4^+CD44^+IL\text{-}21^+$ cells in *CD4-Cre:LepR*$^{+/+}$ and *CD4-Cre:LepR*$^{fl/fl}$ mice 9 days post H1N1 influenza virus infection presented on Fig 4c, WT and *db/db* mice 9 days post NP-KLH/CFA immunization presented on Fig S4e.

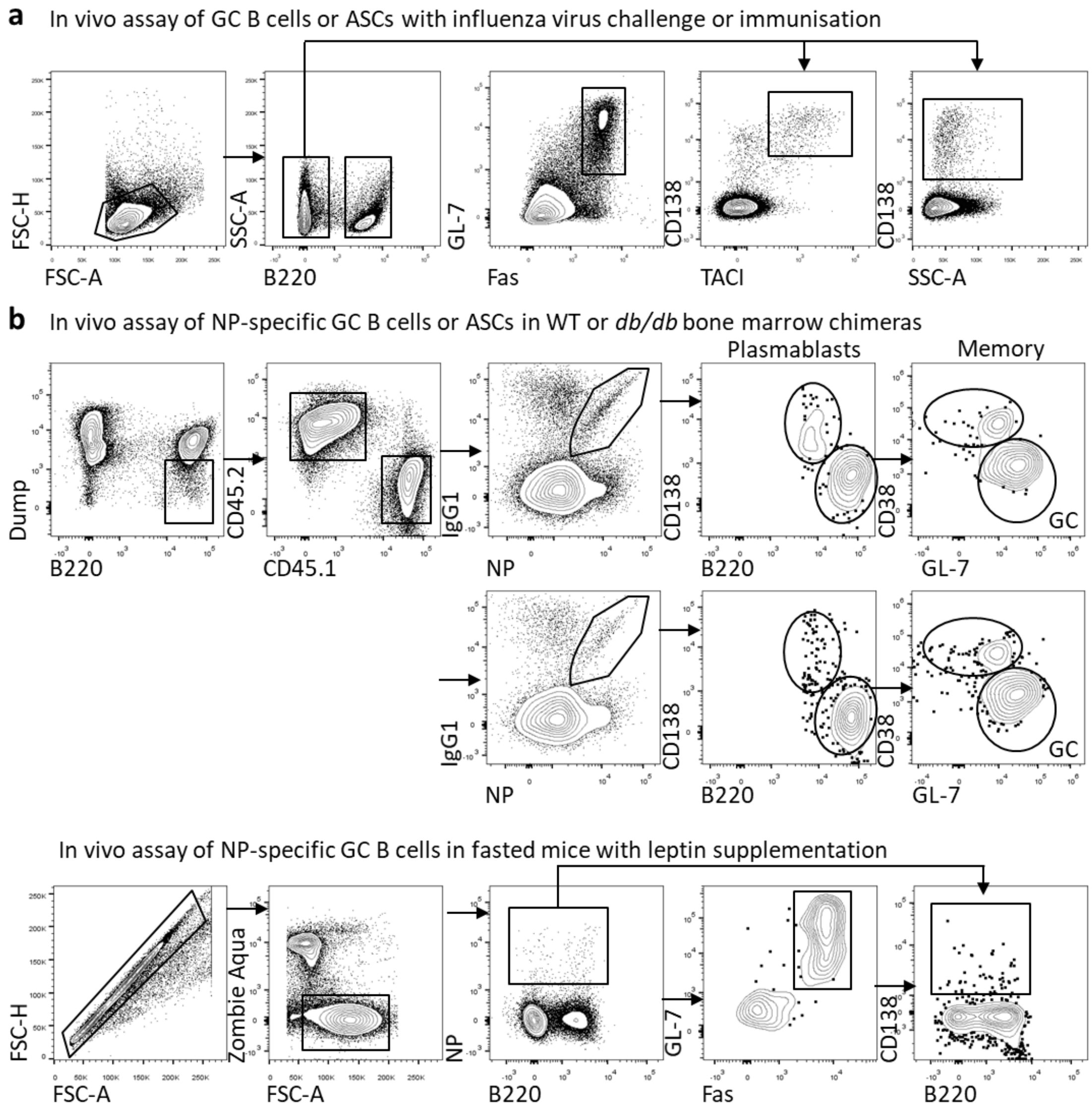

Supplementary Figure 15. **Gating strategies used for flow cytometric analysis of mouse B cells in vivo**

a, Gating strategy to analyze ZA$^-$B220$^+$GL-7$^+$Fas$^+$ germinal centre (GC) B cells, and ZA$^-$B220$^-$CD138^{high} antibody-secreting cells (ASCs) in WT and *db/db* 9 days post H1N1 influenza virus infection presented on Fig 3e, f, *CD4-Cre:LepR*$^{+/+}$ and *CD4-Cre:LepR*$^{fl/fl}$ mice with H1N1 influenza virus infection presented on Fig 4d, e, WT mice with vaccination-challenge model presented on Fig 7d, e, WT and *db/db* mice 9 days post NP-OVA immunization presented on Fig S4c, e, Rag1-/- recipients with mixed CD4++B220+ cells transfer presented on Fig S5b, and *CD4-Cre:LepR*$^{fl/fl}$ mice with human influenza vaccine 20 days post immunization presented on Fig S10d.

b, Gating strategy to analyze NP-specific B220$^+$GL-7$^+$Fas$^+$ GC B cells, and ZA$^-$B220$^-$CD138^{high} ASCs in *Rag1*$^{-/-}$ bone marrow chimeras from WT or *db/db* mice 9 days post NP-OVA immunization presented on Fig S6b, and NP$^+$ GC B and ASCs from CD45.2 recipient mice transferred with CD45.1$^+$ OT-II naïve CD4+ T cells followed by fast and leptin treatment presented on Fig S8d.

Supplementary tables

Supplementary Table 1. Classification of responders and non-responders in adults (18-60 years, n=76) with influenza vaccine

Category	Before Vaccination	After Vaccination
Responder		Any strain: HI change ≥2 fold
Non-Responder		Any strain: HI change <2 fold

Supplementary Table 2. Classification of responders and non-responders in elderly adults (>65 years, n = 83) with influenza vaccine

Category	Before Vaccination	After Vaccination
Responder		Any strain: HI change ≥2 fold
Non-Responder	All strains: HI≤20	Any strain: HI change < 2 fold

Supplementary Table 3. Classification of responders and non-responders Young adults (18-25 years, n = 22) with HBV vaccine

Category	Before Vaccination	After Vaccination
Responder	Titer<100 (mIU/mL)	Titer≥200 (mIU/mL)
Non-Responder	Titer<100 (mIU/mL)	Titer<200 (mIU/mL)

Supplementary Table 4. High cT_{FH} and Low cT_{FH} activity responders in adults (18-60 years, *n* = 76) with influenza vaccine

Category	After Vaccination
High cT_{FH} activity	cT_{FH} change >1 fold
Low cT_{FH} activity	cT_{FH} change ≤ 1 fold

Supplementary Table 5. Demographics of adults with influenza vaccine

Characteristics	Responder (n=71)	Non-responder (n=5)	P-value
Age, year, Median (range)	30 (18-54)	26 (22-40)	
Age, year, Mean±SD	31.0±8.1	28.0±7.2	0.332
H1N1 antibody titer fold change, Median (range)	2.4 (1-61.8)	1 (1.0-1.1)	
H1N1 antibody titer fold change, Mean±SD	5.0±7.9	1.0±0.1	0.001**
H3N2 antibody titer fold change, Median (range)	4 (0.1-64)	1 (1-1.2)	
H3N2 antibody titer fold change, Mean±SD	8.4±12.6	1.0±0.1	0.001**
B strain antibody titer fold change, Median (range)	4 (0.5-68)	1 (0.8-1.2)	
B strain antibody titer fold change, Mean±SD	8.9±12.1	1.0±0.2	<0.001**
T_{FH} fold change, Median (range)	1.7 (0.2-11.7)	0.9 (0.1-1.4)	
T_{FH} fold change, Mean±SD	2.2±1.8	0.9±0.5	0.011*
Leptin (ng/mL), Median (range)	10.5 (1-72.9)	4.2 (2.1-10.2)	
Leptin (ng/mL), Mean±SD	15±14.3	4.8±3.2	0. 030*

Age, antibody titer fold changes, T_{FH} fold changes, and Leptin levels among responder and non-responders were analysed with Mann-Whitney test. *: P value < 0.05; **: P value < 0.01.

Supplementary Table 6. Demographics of elderly adults with influenza vaccine

Characteristics	Responder (n=68)	Non-responder (n=15)	P-value
Age, year, Median (range)	70 (65-88)	73 (65-84)	
Age, year, Mean±SD	70.8±5.4	72.4±4.6	0.138
Gender, male/female	30/38	11/4	
H1N1 antibody titer fold change, Median (range)	4.0 (0.5-256)	2.0 (0.25-2.0)	
H1N1 antibody titer fold change, Mean±SD	18.6±39.2	1.6±0.6	0.015*
H3N2 antibody titer fold change, Median (range)	8.0 (0.5-645)	2.0 (1-4)	
H3N2 antibody titer fold change, Mean±SD	49.3±100.8	2.2±1.2	0.013*
B strain antibody titer fold change, Median (range)	2.0 (0.5-128)	1.0 (0.6-2)	
B strain antibody titer fold change, Mean±SD	12.5±25.7	1.2±0.4	<0.001**
Leptin (ng/mL), Median (range)	15.5 (2.4-91.9)	8.0 (2.5-39.6)	
Leptin (ng/mL), Mean±SD	22.5±18.6	11.3±11.3	<0. 001*

Age, antibody titer fold changes, and Leptin levels among responder and non-responders were analysed with Mann-Whitney test. *: P value < 0.05; **: P value < 0.01.

Supplementary Table 7. Demographics of young adults with HBV vaccine

Characteristics	Responder (n=14)	Non-responder (n=8)	P-value
Age, year, median (range)	20 (20-21)	20 (19-21)	
Age, year, Mean±SD	20.1±0.3	20.0±0.5	>1
Gender, male/female	4/10	8/0	
Before vaccination			
Antibody titer, mIU/mL, Median (range)	11.2 (0-41.4)	0.2 (0-8.2)	
Antibody titer, mIU/mL, Mean±SD	16.3±16.2	1.3±2.8	<0.002**
After vaccination			
Antibody titer, mIU/mL, Median (range)	1000 (409.7-1000)	55.4 (0-198.1)	
Antibody titer, mIU/mL, Mean±SD	841.9±258.3	76.3±87.1	<0.001**
Leptin, ng/mL, Median (range)	16.0 (1.4-41.7)	3.2 (0.3-11.2)	
Leptin, ng/mL, Mean±SD	18.4±12.7	4.4±4.0	0.004**

Age, antibody titer, and Leptin levels among responder and non-responders were analysed with Mann-Whitney test. **: P value < 0.01.

Supplementary Table 8. List of primers used on this study.

	Sense 5'-3'	Antisense 5'-3'
Human q-PCR primers		
CXCR5	TGAAGTTCCGCAGTGACCTGTC	GAGGTGGCATTCTCTGACTCAG
BCL6	CATGCAGAGATGTGCCTCCACA	GAGGTGGCATTCTCTGACTCAG
Il21	CCAAGGTCAAGATCGCCACATG	TGGAGCTGGCAGAAATTCAGGG
PDCD1	AAGGCGCAGATCAAAGAGAGCC	CAACCACCAGGGTTTGGAACTG
ICOS	CCCATAGGATGTGCAGCCTTTG	GGCTGTGTTCACTGCTCTCATG
CD40L	GCGGCACATGTCATAAGTGAGG	GTCCTTGTCTTTTAACGGTCAGC
IL4	CCGTAACAGACATCTTTGCTGCC	GAGTGTCCTTCTCATGGTGGCT
IL17A	CGGACTGTGATGGTCAACCTGA	GCACTTTGCCTCCCAGATCACA
ACTB	CACCATTGGCAATGAGCGGTTC	AGGTCTTTGCGGATGTCCACGT
Mouse q-PCR primers		
Leprb	AAGCCTGAAACATTTGAGCATCTT	ATCGACACTGATTTCTTCTG
Cxcr5	ATCGTCCATGCTGTTCACGCCT	CAACCTTGGCAAAGAGGAGTTCC
Bcl6	CTAAGAGCGCACAAGGCAGTTC	GTCCAGAAGAGGAGCAAAGCCT
Il21	GCCTCCTGATTAGACTTCGTCAC	CAGGCAAAAGCTGCATGCTCAC
Icos	GCAGCTTTCGTTGTGGTACTCC	TGTGTTGACTGCCGCCATGAAC
Cd40l	GAACTGTGAGGAGATGAGAAGGC	TGGCTTCGCTTACAACGTGTGC
Il4	ATCATCGGCATTTTGAACGAGGTC	ACCTTGGAAGCCCTACAGACGA
Il17a	CAGACTACCTCAACCGTTCCAC	TCCAGCTTTCCCTCCGCATTGA
Actb	GCGTGACATCAAAGAGAAGCT	ATGCCACAGGATTCCATACC
Primers for chromatin immunoprecipitation		
Il21 promoter	TGCCGCTGCTTTACTCATTG	GCACCGTCAGCTTTCAGAGA
Primers for luciferase reporter assay		
	CTTGGTACCAAAAAGCATAGTCATCACCC	GCCGGTACCGATCTTACCTTTACA
H1N1 NP viral RNA		
Np	TGTCHTTCCAGGGGCGGGG	GTCAAARGARGGCACGATCGGG
