# Supplementary material for: The metabolic hormone leptin promotes the function of TFH cells and supports vaccine responses
Source: Nat Commun. 2021 May 24;12:3073. doi: 10.1038/s41467-021-23220-x (PMC8144586; doi:10.1038/s41467-021-23220-x)
Supplement: Supplementary file 2 — Reporting Summary [file 41467_2021_23220_MOESM2_ESM.pdf]

nature research

Corresponding author(s): Di Yu, Liwei Lu

Last updated by author(s): Mar 7, 2021

Reporting Summary

Nature Research wishes to improve the reproducibility of the work that we publish. This form provides structure for consistency and transparency in reporting. For further information on Nature Research policies, see our Editorial Policies and the Editorial Policy Checklist.

Statistics

For all statistical analyses, confirm that the following items are present in the figure legend, table legend, main text, or Methods section.

n/a	Confirmed	
☐	☒	The exact sample size (*n*) for each experimental group/condition, given as a discrete number and unit of measurement
☐	☒	A statement on whether measurements were taken from distinct samples or whether the same sample was measured repeatedly
☐	☒	The statistical test(s) used AND whether they are one- or two-sided<br>*Only common tests should be described solely by name; describe more complex techniques in the Methods section.*
☐	☒	A description of all covariates tested
☐	☒	A description of any assumptions or corrections, such as tests of normality and adjustment for multiple comparisons
☐	☒	A full description of the statistical parameters including central tendency (e.g. means) or other basic estimates (e.g. regression coefficient) AND variation (e.g. standard deviation) or associated estimates of uncertainty (e.g. confidence intervals)
☐	☒	For null hypothesis testing, the test statistic (e.g. *F*, *t*, *r*) with confidence intervals, effect sizes, degrees of freedom and *P* value noted<br>*Give P values as exact values whenever suitable.*
☒	☐	For Bayesian analysis, information on the choice of priors and Markov chain Monte Carlo settings
☒	☐	For hierarchical and complex designs, identification of the appropriate level for tests and full reporting of outcomes
☐	☒	Estimates of effect sizes (e.g. Cohen's *d*, Pearson's *r*), indicating how they were calculated

Our web collection on statistics for biologists contains articles on many of the points above.

Software and code

Policy information about availability of computer code

Data collection	BD FACS Diva Software (BD Biosciences v8.0), QuantStudio Real-Time PCR Software (ABI, v1.3).
Data analysis	FlowJo (V10), ImageJ (V1.8), Photoshop CC 2019 and GraphPad Prism (Version 8.0).

For manuscripts utilizing custom algorithms or software that are central to the research but not yet described in published literature, software must be made available to editors and reviewers. We strongly encourage code deposition in a community repository (e.g. GitHub). See the Nature Research guidelines for submitting code & software for further information.

Data

Policy information about availability of data

All manuscripts must include a data availability statement. This statement should provide the following information, where applicable:

- Accession codes, unique identifiers, or web links for publicly available datasets
- A list of figures that have associated raw data
- A description of any restrictions on data availability

The source data of statistics used in main Figures 1-7, and supplementary Figures 1-11 are provided as source data_Statistics.
The raw gel bands for Western blots presented on Figure 5c, Figure 6c-e, and q-PCR gels presented on Figure 5d are provided as source data_Raw gels.
All data are available within the Article and Supplementary Files, or available from the authors upon request.

Field-specific reporting

Please select the one below that is the best fit for your research. If you are not sure, read the appropriate sections before making your selection.

☒ Life sciences ☐ Behavioural & social sciences ☐ Ecological, evolutionary & environmental sciences

For a reference copy of the document with all sections, see nature.com/documents/nr-reporting-summary-flat.pdf

Life sciences study design

All studies must disclose on these points even when the disclosure is negative.

Sample size	Total 3 cohorts are used in this study. 76 healthy adults (aged 18-60 years) with seasonal flu vaccine, 83 healthy adults (aged >65 years) with seasonal flu vaccine, and 22 adults with HBV vaccine are utilized in Figure 1.<br>In vitro culture of human naive CD4+ T cells isolated from healthy donors, n=3-5 per gourp/treatment are used in Fig 2c-g.<br>In mice experiments, n= 4-19 mice per group are used. The exact n=mice utilized per group/treament are noted in each experiment.
Data exclusions	N/A. No data are excluded.
Replication	All data were collected from biological replicates or independent experiments with consistent results.
Randomization	Animals were randomly allocated into either control or experimental groups.
Blinding	Not relevant to the main part of this study. Data collection and analysis were not affected by investigator bias.

Reporting for specific materials, systems and methods

We require information from authors about some types of materials, experimental systems and methods used in many studies. Here, indicate whether each material, system or method listed is relevant to your study. If you are not sure if a list item applies to your research, read the appropriate section before selecting a response.

Materials & experimental systems

n/a	Involved in the study	
☐	☒	Antibodies
☐	☒	Eukaryotic cell lines
☒	☐	Palaeontology and archaeology
☐	☒	Animals and other organisms
☐	☒	Human research participants
☒	☐	Clinical data
☒	☐	Dual use research of concern

Methods

n/a	Involved in the study	
☒	☐	ChIP-seq
☐	☒	Flow cytometry
☒	☐	MRI-based neuroimaging

Antibodies

Antibodies used	Anti-CD45.2-APC (104), anti-CD45.1 BV510 (A20), anti-B220-AF700 (RA3-6B2), anti-GL-7-PE (GL7), anti-Fas-PE-Cy7 (SA367H8), anti-CD138-BV711 (281-2), anti-CD8-AF700 (53-6.7), anti-CD44-PE/Cy7 (IM7), anti-CD62L-BV711 (MEL-14), anti-TACI-PE (8F10), anti-PD-1-BV421 (29F.1A12), anti-IgG1-APC (RMG1-1), anti-IL-17A-FITC (TC11-18H10.1), anti-IFN-g-PE-Cy7 (XMG1.2), anti-IL-4-PE/Dazzle 594 (11B11), anti-CD19-FITC (6D5), biotin anti-mouse CD185 (L138D7). Anti-IgM-BV421 (R6-60.2), anti-CD38-FITC (90/CD38), anti-Bcl6-PE (K112-91), anti-CD4-BV510 (GK1.5) were purchased from BD Pharmingen. Anti-IL-21-PE (mhalx21), anti-Foxp3-FITC (FJK-16s), were purchased from eBioscience.<br>Human antibody for FACS<br>Anti-CD4-BV510 (), anti-CD8-AF700 (), anti-CD25-FITC (), anti-CD127-BV650 (), anti-anti-CCR7-APC/Cy7 (G043H7), anti-CD45RA-PE-Cy7 (HI100), anti-CXCR5-APC (J252D4), anti-PD-1-BV711 (EH12.2H7) anti-human IL-21-PE (eBio3A3-N2 (3A3-N2)).<br>Primary antibodies for Western blot<br>anti-Akt, anti-phospho-Akt (Thr308), anti-Stat3, anti-phospho-Stat3 (Tyr705), anti-mTOR, anti-phospho-mTOR (Ser2448), anti-p70S6K, anti-phospho-p70S6K (Thr389), anti-S6, anti-phospho-S6 (Ser240/244), and anti-β-actin were purchased from Cell Signalling Technology. Anti-Bcl6 (clone: IG191E/A8) were obtained from Biolegend.<br>Primary antibodies for Immunofluorescence staining<br>Alexa Fluor 488 anti-mouse IgM (Biolegend), Brilliant Violet 421 anti-mouse CD4 (Biolegend), Alexa Fluor 555 leptin (Bioss), Brilliant Violet 421 anti-mouse CD19 (Biolegend), Alexa Fluor 488 anti-mouse F4/80 (Biolegend).
Validation	Mouse antibody for FACS<br>anti-CD45.2, (APC, clone 104, Biolegend), PMID: 28841417, PMID: 29136509, PMID: 30013160, PMID: 29802020, PMID: 29155425;<br>anti-CD45.1 (BV510, clone A20, Biolegend), PMID: 29618658, PMID: 30356668, PMID: 29358051, PMID: 28943329, PMID: 31527834;<br>anti-B220 (AF700, clone RA3-6B2, Biolegend), PMID: 30257198, PMID: 29752065, PMID: 31101805, PMID: 31462639;

anti-GL-7 (PE, clone GL7, Biolegend), PMID: 29396162, PMID: 29287996, PMID: 30254309, PMID: 31216480, PMID: 31896754;
anti-Fas (PE-Cy7, clone SA367H8, Biolegend), PMID: 29541074, PMID: 29606496, PMID: 30546010, PMID: 31130952;
anti-CD138 (BV711, clone 281-2, Biolegend), PMID: 31722204, PMID: 32213346;
anti-CD8 (AF700, clone 53-6.7, Biolegend), PMID: 29858012, PMID: 30540933, PMID: 27736323, PMID: 29580144, PMID: 31018134;
anti-CD44 (PE/Cy7, clone IM7, Biolegend), PMID: 28668507, PMID: 28673287, PMID: 28793333, PMID: 29552007, PMID: 29669249;
anti-CD62L (BV711, clone MEL-14, Biolegend), PMID: 30540933, PMID: 30970256, PMID: 28345001, PMID: 32209472, PMID: 28364673;
anti-TACI (PE, clone 8F10, Biolegend), PMID: 30842412, PMID: 23074274;
anti-PD-1 (BV421, clone 29F.1A12, Biolegend), PMID: 30709742, PMID: 30800128, PMID: 29336888, PMID: 30926234, PMID: 28700579;
anti-IgG1 (APC, clone RMG1-1, Biolegend), PMID: 30257198, PMID: 29396162, PMID: 31304630, PMID: 31896754, PMID: 30017584;
anti-IL-17A (FITC, clone TC11-18H10.1, Biolegend), PMID: 29183643, PMID: 29805810, PMID: 29874567, PMID: 29262351, PMID: 29261232;
anti-IFN-g (PE-Cy7, clone XMG1.2, Biolegend), PMID: 28864426, PMID: 29127360, PMID: 29415756, PMID: 29610255, PMID: 29900011;
anti-IL-4 (PE/Dazzle 594, clone 11B11, Biolegend), PMID: 31593700, PMID: 31704965;
anti-CD19 (FITC, clone 6D5, Biolegend), PMID: 29255233, PMID: 29695802, PMID: 29754823, PMID: 30054206, PMID: 30231983;
anti-CD185 (biotin, clone L138D7, Biolegend), PMID: 31216480, PMID: 29743364, PMID: 32049020, PMID: 31527834, PMID: 32783949;
anti-IgM (BV421, clone R6-60.2, BD Pharmingen),
anti-CD38 (FITC, clone 90/CD38, Biolegend), PMID: 30265241, PMID: 30970258, PMID: 31120187, PMID: 29852257, PMID: 32373115;
anti-Bcl6 (PE, clone K112-91, BD Pharmingen),
anti-CD4 (BV510, clone GK1.5, BD Pharmingen),
anti-IL-21 (PE, clone mhalx21, eBioscience), PMID: 25941359, PMID: 28443628;
anti-Foxp3 (FITC, clone FJK-16s, eBioscience), PMID: 19690518, PMID: 26056145, PMID: 30393583, PMID: 31940491;

Human antibody for FACS
anti-CD4 (BV510, clone OKT4, Biolegend), PMID: 28582466, PMID: 29321369, PMID: 29632724, PMID: 31234485, PMID: 30267761;
anti-CD8 (AF700, clone SK1, Biolegend), PMID: 29989547, PMID: 29262349, PMID: 31141680, PMID: 31337064, PMID: 32081864;
anti-CD25 (FITC, clone M-A251, Biolegend), PMID: 29634393;
anti-CD127 (BV650, clone A019D5, Biolegend), PMID: 30221739, PMID: 30127434, PMID: 31175140, PMID: 28414293, PMID: 26295709;
anti-anti-CCR7 (APC/Cy7, clone G043H7, Biolegend), PMID: 30463012, PMID: 29802019, PMID: 31519219, PMID: 31533045, PMID: 28798369;
anti-CD45RA (PE-Cy7, clone HI100, Biolegend), PMID: 29147018, PMID: 29290585, PMID: 30683619, PMID: 31398192, PMID: 30816327;
anti-CXCR5 (APC, clone J252D4, Biolegend), PMID: 28440484, PMID: 31168514, PMID: 30566881, PMID: 32668246;
anti-PD-1 (BV711, clone EH12.2H7, Biolegend), PMID: 28886065, PMID: 29312358, PMID: 29321369, PMID: 29664020, PMID: 30270043;
anti-human IL-21 (PE, clone eBio3A3-N2 (3A3-N2), eBioscience). PMID: 30568034, PMID: 19843932, PMID: 29426881;
Primary antibodies for Western blot
anti-Akt, (Cell Signalling Technology), PMID: 33334822, PMID: 33311440, PMID: 33283987;
anti-phospho-Akt (Thr308), PMID: 33177525, PMID: 31957537, PMID: 32901877;
anti-Stat3 (Cell Signalling Technology), PMID: 32214092, PMID: 32649314;
anti-phospho-Stat3 (Tyr705) (Cell Signalling Technology), PMID: 33283987, PMID: 33182312;
anti-mTOR (Cell Signalling Technology), PMID: 32649314, PMID: 33268783;
anti-phospho-mTOR (Ser2448) (Cell Signalling Technology), PMID: 33268783, PMID: 32545395;
anti-p70S6K (Cell Signalling Technology), PMID: 33268783, PMID: 33289483;
anti-phospho-p70S6K (Thr389) (Cell Signalling Technology), PMID: 30837833; PMID: 31011190;
anti-S6 (Cell Signalling Technology), PMID: 33082139, PMID: 33332560;
anti-phospho-S6 (Ser240/244) (Cell Signalling Technology), PMID: 32934215, PMID: 32930093;
anti-β-actin (Cell Signalling Technology), PMID: 33207194, PMID: 33311440;
anti-Bcl6 (clone: IG191E/A8, Biolegend), PMID: 16899758.
Primary antibodies for Immunofluorescence staining
anti-IgM (Alexa Fluor 488, clone RMM-1, Biolegend), PMID: 30135079, PMID: 30762518;
anti-CD4 (Brilliant Violet 421, clone GK1.5, Biolegend), PMID: 30463976, PMID: 31043609, PMID: 30775090, PMID: 30759395, PMID: 29677517, PMID: 29874567;
anti-CD19 (Brilliant Violet 421, clone 6D5, Biolegend), PMID: 29985390, PMID: 30017352, PMID: 30619263, PMID: 27736323, PMID: 30770250, PMID: 30843878;
anti-F4/80 (Alexa Fluor 488, clone BM8, Biolegend), PMID: 29241547, PMID: 29273735, PMID: 29255242, PMID: 29295986, PMID: 29695802;
Alexa Fluor 555 leptin (Bioss), APPLICATIONS: IF(IHC-P), IF(IHC-F), IF(ICC); REACTIVITY: Human, Mouse, Rat; PREDICTED REACTIVITY: Sheep, Pig

Eukaryotic cell lines

Policy information about cell lines

Cell line source(s)	293T cell line was purchased from the ATCC.
Authentication	293T cell line was authenticated by STR profiling.
Mycoplasma contamination	293T cell line was tested negative for mycoplasma contamination.
Commonly misidentified lines (See ICLAC register)	No commonly misidentified lines were used in this study.

Animals and other organisms

Policy information about studies involving animals; ARRIVE guidelines recommended for reporting animal research

Laboratory animals	Wild-type (WT) C57BL/6, Rag1-/- (B6.129S7-Rag1tm1Mom/J), leptin receptor-deficient (B6.BKS(D)-Leprdb/J, db/db), CD45.1 B6.SJL-Ptprca Pepcb, CD4-Cre (B6.Cg-Tg(Cd4-cre)), Stat3flox (B6.129S1-Stat3tm1Xyfu/J), Leprflox (B6.129P2-Leprtm1Rck/J), OT-II (B6.Cg-Tg (TcraTcrb)425Cbn/J) mice were obtained from Jackson Laboratory and Rictorflox (Rictorflox/flox (Rictortm1.1Klg/SjmJ)) mice were provided by Dr Lilin Ye (Institute of Immunology, Third Military Medical University (Army Medical University))43. Female mice (7-10 weeks of age) on C57BL/6 background were used in this study, and maintained in specific pathogen free animal facilities in the animal facilities of The University of Hong Kong, The Australian National University, Renji Hospital, Shanghai Jiao Tong University School of Medicine.
Wild animals	No wild animals were used in this study.
Field-collected samples	No field-collected samples were used in this study.
Ethics oversight	All experiments were performed under the animal welfare guidelines under approved protocols of The University of Hong Kong, The Australian National University and Shanghai Jiao Tong University.

Note that full information on the approval of the study protocol must also be provided in the manuscript.

Human research participants

Policy information about studies involving human research participants

Population characteristics	Two cohorts of influenza vaccination were examined. Elderly adult healthy volunteers (n=83, > 65 years old) were recruited for the vaccination of 2008/2009 seasonal influenza vaccine (Seqirus, Australia). Age: 18-54 years old.<br>Adult healthy volunteers (n=76, 18-60 years old) were recruited for the vaccination with trivalent or quadrivalent seasonal influenza vaccine (The Cambridge BioResource, Cambridge, UK, Peter Doherty Institute for Infection and Immunity, University of Melbourne, Australia). Age: 65-88 years old. Gender: male 41, female: 42.<br>Young adults (n=22, 18-25 years old) were recruited for the vaccination of Engerix-B® (Hepatitis B surface antigen recombinant (yeast) vaccine, GSK) in Peking University People's Hospital (Beijing, China). Age: 19-21 years old. Gender: male 12, female: 10.
Recruitment	Healthy participants were provided written informed consent according to the ethics approvals from institutions.
Ethics oversight	Ethics approvals were approved by the Monash University Human Research Ethics Committee (elderly with flu vaccine), Lisbon Academic Medical Center Ethics Committee (adults with flu vaccine), the University of Melbourne Human Ethics Committee (adults with flu vaccine), and Peking University People's Hospital Ethics Committee (adults with HBV vaccine), and the Ethical Committee of Renji Hospital (healthy individuals to donate blood) accordingly.

Note that full information on the approval of the study protocol must also be provided in the manuscript.

Flow Cytometry

Plots

Confirm that:

- [ ] The axis labels state the marker and fluorochrome used (e.g. CD4-FITC).
- [x] The axis scales are clearly visible. Include numbers along axes only for bottom left plot of group (a 'group' is an analysis of identical markers).
- [x] All plots are contour plots with outliers or pseudocolor plots.
- [x] A numerical value for number of cells or percentage (with statistics) is provided.

Methodology

Sample preparation	Human blood samples were isolated with the Ficoll-Paque, washed with PBS, filtered with mesh, and suspend with 10% FBS RMPI 1640. Mouse single-cell suspensions from spleens and draining lymph nodes filtered with mesh, and suspend with 10% FBS RMPI 1640.<br>1x10^6 cells single cell suspension were stained with surface antibodies. For intracellular cytokine staining, cells were stimulated with PMA (Sigma-Aldrich), Ionomycin (Sigma-Aldrich), Monensin (Biolegend), or Brefeldin A (BD Pharmingen) for 5 hours and stained with the Fixation/Permeabilization Solution Kit (BD Pharmingen). Intracellular staining of Bcl6 and Foxp3 was performed using the Foxp3/Transcription factor staining set (eBioscience). 7-AAD (7-Aminoactinomycin D) (Thermo Fisher), PI (Propidium Iodide) (Biolegend) and Zombie Aqua™ Fixable Viability Kit (Biolegend) were used to distinguish live/dead cells.
Instrument	BD FACS Aria III celll sorter, BD FACS SORP X-20 analyzer.
Software	Data collection: BD FACS Diva Software (BD Biosciences v8.0);<br>Data analysis: FlowJo_V10.
Cell population abundance	For cell sorting: the purity of human naive CD4+CD25-CD45RA+CCR7+ (95%) and mouse CD4+CD25-CD62L+CD44+ (>95%) for in vitro culture.<br>Other cell population abundance were variable due to the different gating strategies.
Gating strategy	The gating strategy of human and mouse naive CD4+ T cell sorting, cultured TFH cells analysis, in vivo assay of TFH cells, B cells were provided in Supplementary Fig S12-15 accordingly.

☒ Tick this box to confirm that a figure exemplifying the gating strategy is provided in the Supplementary Information.
