# Supplementary material for: The metabolic hormone leptin promotes the function of TFH cells and supports vaccine responses
Source: Nat Commun. 2021 May 24;12:3073. doi: 10.1038/s41467-021-23220-x (PMC8144586; doi:10.1038/s41467-021-23220-x)
Supplement: Supplementary file 3 — Source Data [file 41467_2021_23220_MOESM3_ESM.zip › Source Data/Source data_Western blot and PCR raw gels.pptx]

## Slide 1
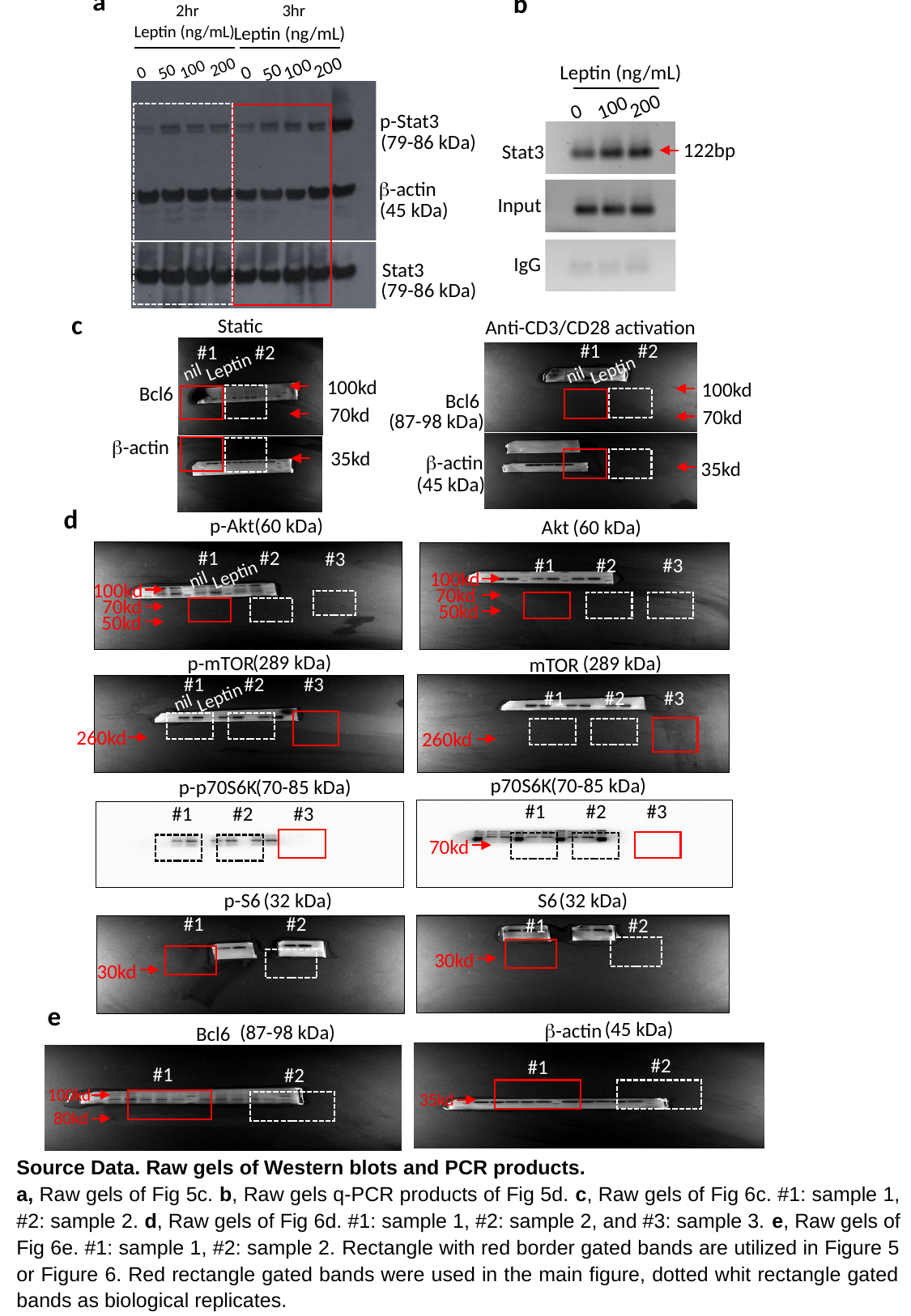

a
3hr
2hr
Leptin (ng/mL)
200
100
50
0
Leptin (ng/mL)
200
100
50
0
p-Stat3
(79-86 kDa)
β-actin
(45 kDa)
Stat3
(79-86 kDa)
b
Leptin (ng/mL)
200
100
0
122bp
Stat3
Input
IgG
c
Static
#1
#2
100kd
Bcl6
70kd
Leptin
nil
b-actin
35kd
Anti-CD3/CD28 activation
#1
#2
100kd
Bcl6
70kd
35kd
β-actin
Leptin
nil
(87-98 kDa)
(45 kDa)
d
p-Akt
(60 kDa)
Akt
(60 kDa)
#1
#2
#3
100kd
70kd
50kd
#1
#2
#3
Leptin
nil
100kd
70kd
50kd
(289 kDa)
p-mTOR
(289 kDa)
mTOR
#1
#2
#3
260kd
#1
#2
#3
260kd
Leptin
nil
p70S6K
(70-85 kDa)
p-p70S6K
(70-85 kDa)
#1
#2
#3
70kd
#1
#2
#3
p-S6
(32 kDa)
S6
(32 kDa)
#1
#2
30kd
#1
#2
30kd
e
(45 kDa)
β-actin
#2
#1
35kd
(87-98 kDa)
Bcl6
#1
#2
100kd
80kd
Source Data. Raw gels of Western blots and PCR products.
a, Raw gels of Fig 5c. b, Raw gels q-PCR products of Fig 5d. c, Raw gels of Fig 6c. #1: sample 1, #2: sample 2. d, Raw gels of Fig 6d. #1: sample 1, #2: sample 2, and #3: sample 3. e, Raw gels of Fig 6e. #1: sample 1, #2: sample 2. Rectangle with red border gated bands are utilized in Figure 5 or Figure 6. Red rectangle gated bands were used in the main figure, dotted whit rectangle gated bands as biological replicates.
